# Amphipathic α-Helices in Apolipoproteins Are Crucial to the Formation of Infectious Hepatitis C Virus Particles

**DOI:** 10.1371/journal.ppat.1004534

**Published:** 2014-12-11

**Authors:** Takasuke Fukuhara, Masami Wada, Shota Nakamura, Chikako Ono, Mai Shiokawa, Satomi Yamamoto, Takashi Motomura, Toru Okamoto, Daisuke Okuzaki, Masahiro Yamamoto, Izumu Saito, Takaji Wakita, Kazuhiko Koike, Yoshiharu Matsuura

**Affiliations:** 1 Department of Molecular Virology, Research Institute for Microbial Diseases, Osaka University, Osaka, Japan; 2 Department of Infection Metagenomics, Research Institute for Microbial Diseases, Osaka University, Osaka, Japan; 3 DNA-Chip Developmental Center for Infectious Diseases, Research Institute for Microbial Diseases, Osaka University, Osaka, Japan; 4 Department of Immunoparasitology, Research Institute for Microbial Diseases, Osaka University, Osaka, Japan; 5 Laboratory of Molecular Genetics, Institute of Medical Science, University of Tokyo, Tokyo, Japan; 6 Department of Virology II, National Institute of Infectious Diseases, Tokyo, Japan; 7 Department of Gastroenterology, Graduate School of Medicine, University of Tokyo, Tokyo, Japan; The Scripps Research Institute, United States of America

## Abstract

Apolipoprotein B (ApoB) and ApoE have been shown to participate in the particle formation and the tissue tropism of hepatitis C virus (HCV), but their precise roles remain uncertain. Here we show that amphipathic α-helices in the apolipoproteins participate in the HCV particle formation by using zinc finger nucleases-mediated apolipoprotein B (ApoB) and/or ApoE gene knockout Huh7 cells. Although Huh7 cells deficient in either ApoB or ApoE gene exhibited slight reduction of particles formation, knockout of both ApoB and ApoE genes in Huh7 (DKO) cells severely impaired the formation of infectious HCV particles, suggesting that ApoB and ApoE have redundant roles in the formation of infectious HCV particles. cDNA microarray analyses revealed that ApoB and ApoE are dominantly expressed in Huh7 cells, in contrast to the high level expression of all of the exchangeable apolipoproteins, including ApoA1, ApoA2, ApoC1, ApoC2 and ApoC3 in human liver tissues. The exogenous expression of not only ApoE, but also other exchangeable apolipoproteins rescued the infectious particle formation of HCV in DKO cells. In addition, expression of these apolipoproteins facilitated the formation of infectious particles of genotype 1b and 3a chimeric viruses. Furthermore, expression of amphipathic α-helices in the exchangeable apolipoproteins facilitated the particle formation in DKO cells through an interaction with viral particles. These results suggest that amphipathic α-helices in the exchangeable apolipoproteins play crucial roles in the infectious particle formation of HCV and provide clues to the understanding of life cycle of HCV and the development of novel anti-HCV therapeutics targeting for viral assembly.

## Author Summary

*In vitro* systems have been developed for the study of hepatitis C virus (HCV) infection and have revealed many details of the life cycle of HCV. Apolipoprotein B (ApoB) and ApoE have been shown to play crucial roles in the particle formation of HCV, based on data obtained by siRNA-mediated gene knockdown and overexpression of the proteins. However, precise roles of the apolipoproteins in HCV assembly have not been elucidated yet. In this study, we show that infectious particle formation of HCV in Huh7 cells was severely impaired by the knockout of both ApoB and ApoE genes by artificial nucleases, and this reduction was cancelled by the expression of not only ApoE, but also other exchangeable apolipoproteins, including ApoA1, ApoA2, ApoC1, ApoC2 and ApoC3. In addition, expression of amphipathic α-helices in the exchangeable apolipoproteins restored the infectious particle formation in the double-knockout cells through an interaction with viral particles. These results provide clues to the understanding of life cycle of HCV and the development of novel antivirals to HCV.

## Introduction

More than 160 million individuals worldwide are infected with hepatitis C virus (HCV), and cirrhosis and hepatocellular carcinoma induced by HCV infection are life-threatening diseases [1]. Current standard therapy combining peg-interferon (IFN), ribavirin (RBV) and a protease inhibitor has achieved a sustained virological response (SVR) in over 80% of individuals infected with HCV genotype 1 [2]. In addition, many antiviral agents targeting non-structural proteins and host factors involved in HCV replication have been applied in clinical trials [3], [4].

*In vitro* systems have been developed for the study of HCV infection and have revealed many details of the life cycle of HCV. By using pseudotype particles bearing HCV envelope proteins and RNA replicon systems, many host factors required for entry and RNA replication have been identified, respectively [5], [6]. In addition, development of a robust *in vitro* propagation system of HCV based on the genotype 2a JFH1 strain (HCVcc) has gradually clarified the mechanism of assembly of HCV particles [7], [8]. It has been shown that the interaction of NS2 protein with structural and non-structural proteins facilitates assembly of the viral capsid and formation of infectious particles at the connection site between the ER membrane and the surface of lipid droplets (LD) [9]. On the other hand, very low density lipoprotein (VLDL) associated proteins, including apolipoprotein B (ApoB), ApoE, and microsomal triglyceride transfer protein (MTTP), have been shown to play crucial roles in the formation of infectious HCV particles [10]–[12]. Generally, ApoA, ApoB, ApoC and ApoE bind the surface of lipoprotein through the interaction between amphipathic α-helices and ER-derived membrane [13], [14]. This binding of apolipoproteins enhances the stability and hydrophilicity of lipoprotein. However, the specific roles played by the apolipoproteins in HCV particle formation are controversial. Gastaminza et al. demonstrated that ApoB and MTTP are cellular factors essential for an efficient assembly of infectious HCV particles [10]. However, studies by other groups demonstrated that ApoE is a major determinant of the infectivity and particle formation of HCV, and the ApoE fraction is highly enriched with infectious particles [11]. In addition, Mancone et al. showed that ApoA1 is required for production of infectious particles of HCV [15]. However, the evidence of the involvement of apolipoproteins in HCV particle formation is dependent on knockdown data and exogenous expression of the apolipoproteins, and thus the precise mechanisms of participation of the apolipoproteins in HCV assembly have not been elucidated [10], [11], [16].

Recently, several novel genome editing techniques have been developed, including methods using zinc finger nucleases (ZFN), transcription activator like-effector nucleases (TALEN) and CRISPR/Cas9 systems [17]–[19]. DNA double strand breaks (DSBs) induced by these artificial nucleases can be repaired by error-prone non-homologous end joining (NHEJ), resulting in mutant mice or cell lines carrying deletions, insertions, or substitutions at the cut site. To clarify the detailed function of gene family with redundant functions, the generation of animals or cell lines carrying multiple mutated genes may be essential.

In this study, Huh7 cell lines deficient in both ApoB and ApoE genes were established by using ZFNs and revealed that ApoB and ApoE redundantly participate in the formation of infectious HCV particles. Interestingly, the expression of other exchangeable apolipoproteins, i.e., ApoA1, ApoA2, ApoC1, ApoC2 and ApoC3, facilitated HCV assembly in ApoB and ApoE double-knockout cells. In addition, the expression of amphipathic α-helices in the exchangeable apolipoproteins restored the production of infectious particles in the double-knockout cells through an interaction with viral particles.

## Results

### Several apolipoproteins participate in the production of infectious viral particles

**Figure 1:**
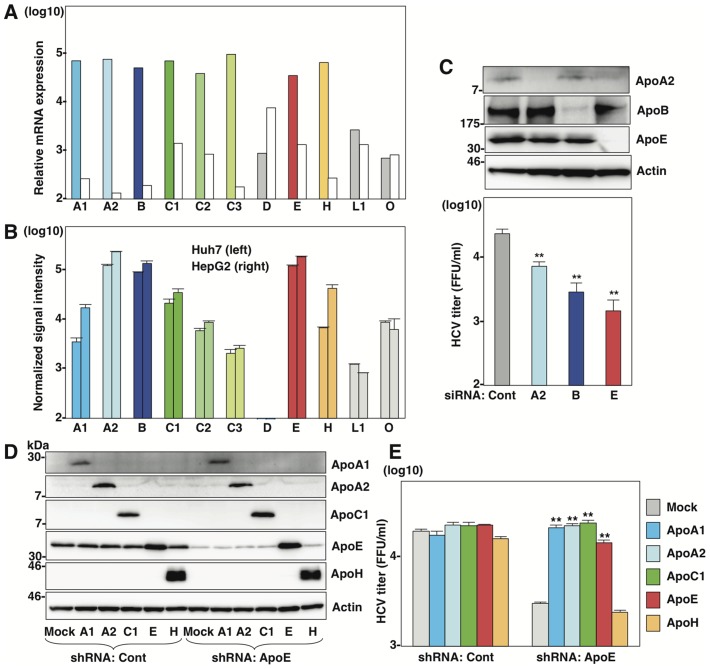
Several apolipoproteins participate in HCV propagation. (A) Relative mRNA expression of the apolipoproteins in the liver tissues (left columns) was determined using the NextBio Body Atlas application. The median expression (right columns) was calculated across all 128 human tissues from 1,068 arrays using the Affymetrix GeneChip Human Genome U133 Plus 2.0 Array. mRNA expression for each gene was log10 transformed. (B) Log10 transformed, normalized signal intensity of the apolipoproteins in Huh7 (left columns) and HepG2 (right columns) cells were extracted from previously published expression microarray dataset GSE32886. (C) Huh7 cells infected with HCVcc at an MOI of 1 at 6 h post-transfection with siRNAs targeting ApoA2 (A2), ApoB (B), ApoE (E) and control (Cont), and expression levels of apolipoproteins (upper panel) and infectious titers in the culture supernatants (lower panel) were determined by immunoblotting and a focus-forming assay at 72 h post-infection, respectively. (D) ApoA1, ApoA2, ApoC1, ApoE and ApoH were exogenously expressed in control and ApoE-knockdown Huh7 cells by lentiviral vectors. Expressions of the apolipoproteins were determined by immunoblotting analysis. (E) Infectious titers in the culture supernatants of control and ApoE-knockdown Huh7 cells expressing the apolipoproteins were determined by focus-forming assay at 72 h post-infection. In all cases, asterisks indicate significant differences (*, P<0.05; **, P<0.01) versus the results for control cells.

First, we compared expression levels of apolipoproteins between hepatocyte and hepatic cancer cell lines including Huh7 and HepG2 cells (Fig. 1A and B). The web-based search engine NextBio (NextBio, Santa Clara, CA) revealed that ApoB, ApoH and the exchangeable apolipoproteins ApoA1, ApoA2, ApoC1, ApoC2, ApoC3, and ApoE are highly expressed in human liver tissues (Fig. 1A). On the other hand, the expressions of ApoA1, ApoC1, ApoC2, ApoC3 and ApoH in hepatic cancer cell lines were suppressed compared to those in hepatocytes (Fig. 1B). To examine the roles of apolipoproteins in the formation of infectious HCV particles, the effects of knockdown of ApoA2, ApoB and ApoE on the infectious particle production in the supernatants were determined in Huh7 cells by focus forming assay (Fig. 1C). The transfection of siRNAs targeting to ApoA2, ApoB and ApoE significantly suppressed the production of infectious HCV particles. This inhibitory effect is well consistent with the high level of expression of these apolipoproteins in the hepatic cancer cell lines, suggesting that the apolipoproteins involved in HCV assembly are dependent on the expression pattern in hepatic cancer cell lines, including Huh7 cells [20]. Therefore, we examined the effects of exogenous expression of the apolipoproteins highly expressed in the liver tissues on the infection of HCV in the stable ApoE-knockdown Huh7 cells (Fig. 1D). In contrast to the control-knockdown cells, expression of not only ApoE but also ApoA1, ApoA2, and ApoC1 rescued the infectious particle formation in the ApoE-knockdown cells (Fig. 1E), suggesting that various exchangeable apolipoproteins participate in the efficient production of infectious HCV particles.

### ApoB and ApoE have a redundant role in HCV particle formation

**Figure 2:**
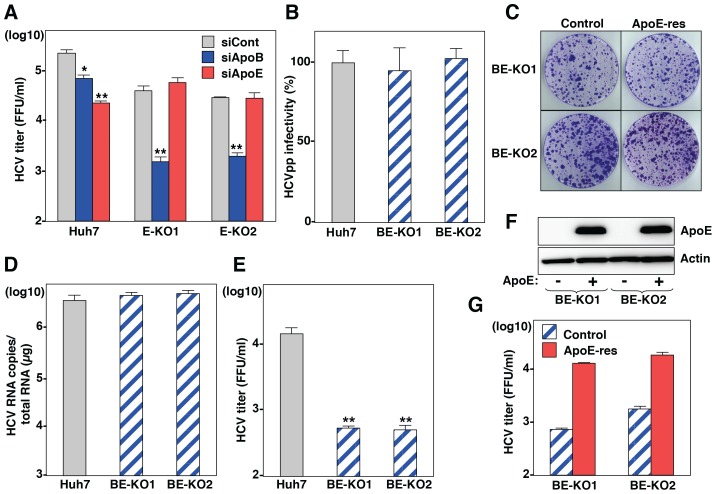
ApoB and ApoE redundantly participate in the formation of infectious HCV particles. (A) Huh7 and E-KO1 cells were infected with HCVcc at an MOI of 1 at 6 h post-transfection with siRNAs targeting ApoB or ApoE, and infectious titers in the culture supernatants were determined by focus-forming assay at 72 h post-infection. (B) HCVpp were inoculated into Huh7, BE-KO1 and BE-KO2 cells, and luciferase activities were determined at 48 h post-infection. (C) A subgenomic HCV RNA replicon of the JFH1 strain was electroporated into BE-KO1 and BE-KO2 cells with/without expression of ApoE by lentiviral vector (ApoE-res), and the colonies were stained with crystal violet at 31 days post-electroporation after selection with 400 µg/ml of G418. Huh7, BE-KO1 and BE-KO2 cells were infected with HCVcc at an MOI of 1, and intracellular HCV RNA (D) and infectious titers in the supernatants (E) were determined at 72 h post-infection by qRT-PCR and focus-forming assay, respectively. (F) Exogenous expression of ApoE in BE-KO1 and BE-KO2 cells by lentiviral vector was determined by immunoblotting analysis. (G) Infectious titers in the culture supernatants of BE-KO1 (gray bars) and ApoE-res cells (red bars) infected with HCVcc at an MOI of 1 were determined at 72 h post-infection by focus-forming assay.

To obtain more convincing data on the involvement of apolipoproteins in the production of infectious HCV particles, we established knockout (KO) Huh7 cells deficient in either ApoB (B-KO1 and B-KO2) or ApoE (E-KO1 and E-KO2) by using ZFN (Figure S1). Deficiencies of ApoB or ApoE expression in these cell lines were confirmed by ELISA and immunoblotting analyses (Figure S1). First, we examined the roles of ApoB and ApoE on the entry and RNA replication of HCV by using HCV pseudotype particles (HCVpp) and subgenomic replicon (SGR) of the JFH1 strain, respectively. The B-KO and E-KO cell lines exhibited no significant effect on the infectivity of HCVpp and the colony formation of SGR (Figure S2A and Figure S2B), suggesting that ApoB and ApoE are not involved in the entry and replication processes of HCV. To examine the role of ApoB and ApoE in the propagation of HCV, HCVcc was inoculated into parental, B-KO and E-KO cell lines at an MOI of 1, and intracellular viral RNA and infectious titers in the supernatants were determined (Figure S2C and Figure S2D). Although RNA replication and infectious particle formation in B-KO cells upon infection with HCV were comparable with those in parental Huh7 cells, E-KO cells exhibited slight reduction of particle formation, and the expression of ApoE in E-KO cells rescued infectious particle formation (Figure S2C, Figure S2D, Figure S2E). Next, to examine the redundant role of ApoB, the effect of knockdown of ApoB on HCV assembly was determined in parental and E-KO Huh7 cell lines (Fig. 2A). Knockdown of ApoB in E-KO cells resulted in a more efficient reduction of infectious particle production than that in parental Huh7 cells, suggesting that ApoB and ApoE have a redundant role in the formation of infectious HCV particles.

To further confirm the redundant role of ApoB and ApoE in the HCV life cycle, especially in the particle formation, 2 clones of ApoB and ApoE double-knockout (BE-KO1 and BE-KO2) Huh7 cells were established by ZFNs (Figure S3A and Figure S3B). The lack of ApoB and ApoE expressions was confirmed by immunoblotting and ELISA analyses (Figure S3C, Figure S3D, Figure S3E). The BE-KO cell lines also exhibited no significant effect on the infectivity of HCVpp (Fig. 2B) and the colony formation of SGR (Fig. 2C). Next, we examined the redundant role of ApoB and ApoE on the propagation of HCVcc. Upon infection with HCVcc at an MOI of 1, infectious titers in the supernatants of BE-KO1 and BE-KO2 cells were 50 to 100 times lower than those of parental Huh7 cells at 72 h post-infection, while the level of intracellular RNA replication was comparable (Fig. 2D and E). In addition, exogenous expression of ApoE in BE-KO (ApoE-res) cells rescued the production of infectious viral particles to levels comparable to those in parental Huh7 cells (Fig. 2F and G), suggesting that ApoB and ApoE redundantly participate in the particle formation of HCV.

### MTTP participates in HCV particle formation through the maturation of ApoB

**Figure 3:**
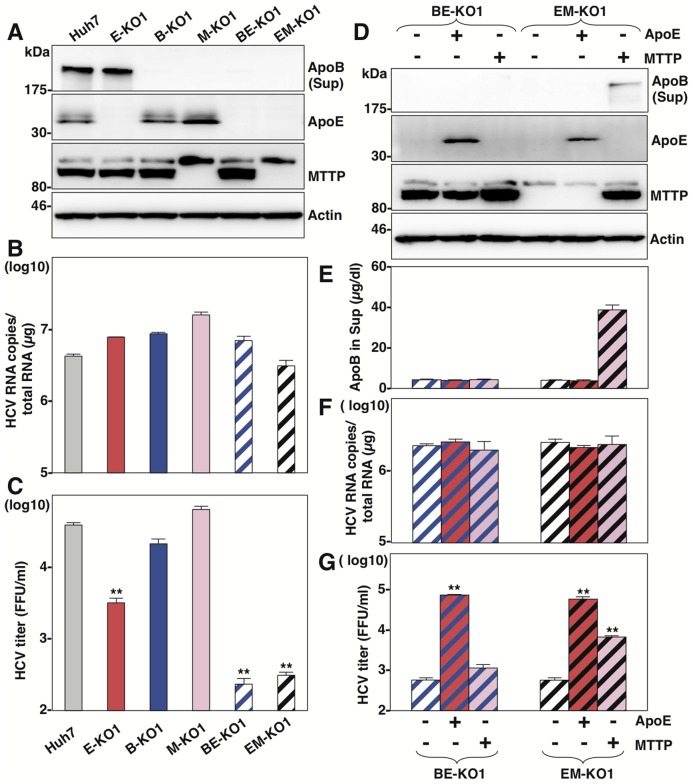
MTTP participates in the formation of infectious HCV particles through the maturation of ApoB. (A) Expressions of ApoB, ApoE and MTTP in Huh7, B-KO1, M-KO1, E-KO1, BE-KO1 and EM-KO1 cells were determined by immunoblotting analysis. Cells were infected with HCVcc at an MOI of 1, and intracellular HCV RNA (B) and infectious titers in the supernatants (C) were determined at 72 h post-infection by qRT-PCR and focus-forming assay, respectively. The expressions of ApoB, ApoE and MTTP in BE-KO1 and EM-KO1 cells with/without expression of ApoE or MTTP by lentiviral vector were determined by immunoblotting (D) and ELISA (E). Cells were infected with HCVcc at an MOI of 1, and intracellular HCV RNA (F) and infectious titers in the supernatants (G) were determined at 72 h post-infection by qRT-PCR and focus-forming assay, respectively.

It is difficult to determine the roles of ApoB in the particle formation of HCV, because ApoB is too large (550 kDa) to obtain cDNA for expression. However, previous reports have shown that expression of MTTP facilitates the secretion of ApoB [21]. To further clarify the roles of ApoB in the life cycle of HCV, we established knockout Huh7 cell lines deficient in MTTP (M-KO1 and M-KO2) and in both ApoE and MTTP (EM-KO1 and EM-KO2) by using the ZFN and CRISPR/Cas9 system (Figure S4A and Figure S4E). The lack of MTTP, ApoB and ApoE expressions was confirmed by immunoblotting and ELISA analyses (Figure Figure S4B, Figure S4C, Figure S4D, Figure S4F, Figure S4G, Figure S4H). As previously reported, the secretion of ApoB was completely abrogated in M-KO and EM-KO cells, while the mRNA levels of ApoB were comparable among Huh7, M-KO and EM-KO cells (Figure S4I). To examine the roles of MTTP in the assembly of HCV through the secretion of ApoB, HCVcc was inoculated into the Huh7, B-KO, M-KO, E-KO, BE-KO and EM-KO cell lines at an MOI of 1, and intracellular HCV genomes and infectious titers in the supernatants were determined (Fig. 3A–C). Although intracellular RNA replication in M-KO and EM-KO cells was comparable with that in Huh7, B-KO, E-KO and BE-KO cells (Fig. 3B), infectious titers in the supernatants of EM-KO cells were severely impaired as seen in BE-KO cells, while those of M-KO cells were comparable to those of parental Huh7cells (Fig. 3C), suggesting that MTTP participates in the HCV assembly through the regulation of ApoB secretion. To further confirm the roles of MTTP in HCV assembly through ApoB secretion, the effects of exogenous expression of MTTP in EM-KO cells on the infectious particle formation of HCV were determined. Immunoblotting and ELISA analyses revealed that exogenous expression of MTTP rescued the secretion of ApoB into the supernatants of EM-KO cells (Fig. 3D and E), while expression of ApoE or MTTP in both BE-KO and EM-KO cells exhibited no effect on the intracellular RNA replication (Fig. 3F). Although exogenous expression of ApoE rescued the infectious particle formation of HCV in both BE-KO and EM-KO cells, expression of MTTP rescued the particle formation in EM-KO cells but not in BE-KO cells (Fig. 3G), supporting the notion that MTTP plays a crucial role in the HCV assembly through the maturation of ApoB.

### Exchangeable apolipoproteins redundantly participate in the assembly of infectious HCV particles

**Figure 4:**
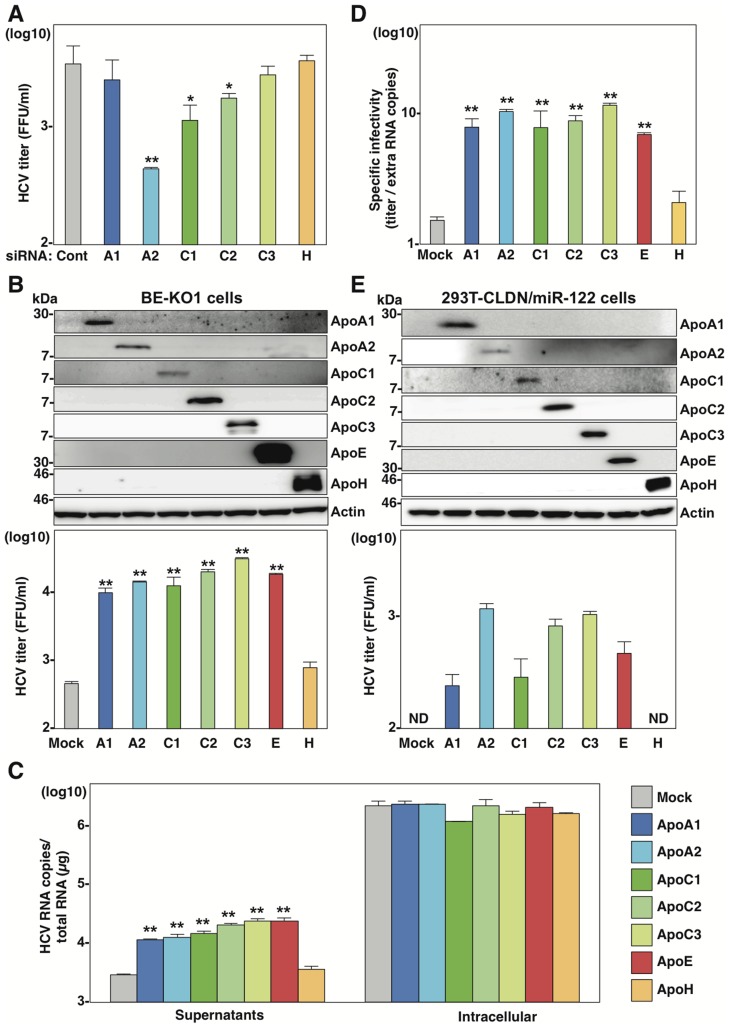
Exchangeable apolipoproteins redundantly participate in the formation of infectious HCV particles. (A) BE-KO1 cells infected with HCVcc at an MOI of 1 at 6 h post-transfection with siRNAs targeting ApoA1 (A1), ApoA2 (A2), ApoC1 (C1), ApoC2 (C2), ApoC3 (C3) and ApoH (H) and infectious titers in the culture supernatants were determined by focus-forming assay at 72 h post-infection. (B) ApoA1, ApoA2, ApoC1, ApoC2, ApoC3, ApoE and ApoH were exogenously expressed in BE-KO1 cells by infection with lentiviral vectors, and then infected with HCVcc at an MOI of 1. Expression of the apolipoproteins was determined by immunoblot analysis (upper), and infectious titers in the culture supernatants were determined at 72 h post-infection by focus-forming assay (lower). (C) Extracellular and intracellular HCV RNA in BE-KO1 cells expressing apolipoproteins and infected with HCVcc were determined at 72 h post-infection by qRT-PCR. (D) Specific infectivity was calculated as extracellular infectious titers/extracellular HCV RNA copies in BE-KO1 cells expressing apolipoproteins at 72 h post-infection. (E) 293T cells stably expressing CLDN1 and miR-122 (293T-CLDN/miR-122 cells) were infected with the lentiviral vectors, and the expressions of the apolipoproteins were determined by immunoblot analysis (upper). These cells were infected with HCVcc at an MOI of 1, and infectious titers in the supernatants were determined at 72 h post-infection by focus-forming assay (lower). In all cases, asterisks indicate significant differences (*, P<0.05; **, P<0.01) versus the results for control cells.

Next, to examine the roles played in HCV particles formation by other apolipoproteins highly expressed in the liver (Fig. 1A), the expressions of ApoA1, ApoA2, ApoC1, ApoC2, ApoC3 and ApoH in BE-KO1 cells were suppressed by siRNAs (Fig. 4A and Figure S5). While knockdown of ApoA1, ApoC3 and ApoH exhibited no effect, that of ApoA2, ApoC1 and ApoC2 significantly inhibited the release of infectious particles, which was consistent with the expression pattern of endogenous apolipoproteins except for ApoH in Huh7 cells (Fig. 1B), suggesting that not only ApoB and ApoE but also other exchangeable apolipoproteins participate in HCV particle formation. To confirm the redundant role of these apolipoproteins on the infectious particle formation, the effects of exogenous expression of these apolipoproteins on the propagation of HCVcc in BE-KO1 cells were determined. ApoA1, ApoA2, ApoC1, ApoC2, ApoC3, ApoE and ApoH were expressed by lentiviral vector in BE-KO1 cells (Fig. 4B upper panel). The expressions of ApoA1, ApoA2, ApoC1, ApoC2, ApoC3 and ApoE but not of ApoH enhanced extracellular HCV RNA, while they exhibited no effect on intracellular HCV RNA (Fig. 4C). In addition, the expressions of these exchangeable apolipoproteins enhanced the infectious particle formation in the supernatants of BE-KO1 cells (Fig. 4B lower panel). On the other hand, the expression of nonhepatic apolipoproteins, including ApoD, ApoL1, and ApoO, exhibited no effect on HCV particle formation in BE-KO1 cells (Figure S6). These results suggest that exogenous expression of not only the ApoE but also the ApoA and ApoC families can compensate for the impairment of HCV particle formation in BE-KO1 cells. Interestingly, specific infectivity (infectious titers/viral RNA levels in supernatants) was also enhanced by the expression of ApoA1, ApoA2, ApoC1, ApoC2, ApoC3 and ApoE, suggesting that these apolipoproteins participate in the infectious but not non-infectious particle formation of HCV (Fig. 4D). Previous reports have suggested that the expressions of Claudin1 (CLDN1), miR-122 and ApoE facilitate the production of infectious particles in nonhepatic 293T cells [16]. Therefore, the effects of exogenous expression of exchangeable apolipoproteins on particle formation were examined in 293T cells expressing CLDN1 and miR-122 (293T-CLDN/miR-122 cells). Exogenous expression of ApoA1, ApoA2, ApoC1, ApoC2, ApoC3 and ApoE, but not of ApoH by lentiviral vector facilitated the production of infectious HCV particles in 293T-CLDN/miR-122 cells (Fig. 4E). On the other hand, the expression of ApoE exhibited no effect on the propagation of Japanese encephalitis virus (JEV) and dengue virus (DENV) (Figure S7) in BE-KO1 cells. These results suggest that the exchangeable apolipoproteins and ApoB redundantly and specifically participate in the formation of HCV particles.

**Figure 5:**
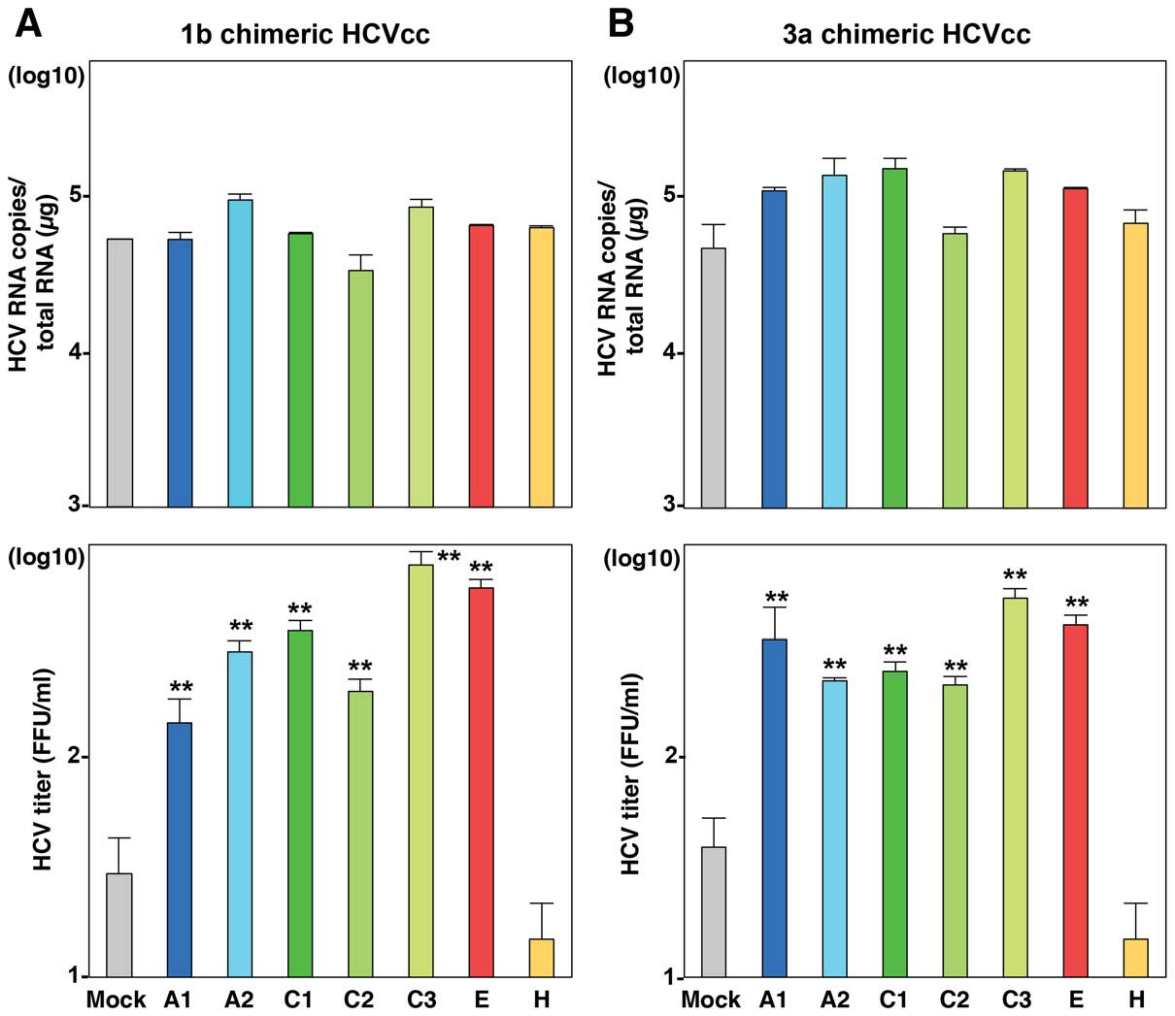
Exchangeable apolipoproteins participate in the formation of infectious HCV particles of genotype 1 and 3. ApoA1, ApoA2, ApoC1, ApoC2, ApoC3, ApoE and ApoH were exogenously expressed in BE-KO1 cells by infection with lentiviral vectors, and then infected with genotype 1b and 3a chimeric HCVcc, TH/JFH1 (A) and S310/JFH1 (B) at an MOI of 0.5. Intracellular HCV RNA and infectious titers in the culture supernatants were determined at 72 h post-infection by qRT-PCR (upper) and focus-forming assay (lower). Asterisks indicate significant differences (**, P<0.01) versus the results for control cells.

To examine the role of exchangeable apolipoproteins in the formation of other genotypes of HCV, the effect of exogenous expression of these apolipoproteins on the propagation of genotype 1b and 3a chimeric HCVcc, TH/JFH1 and S310/JFH1 viruses in BE-KO1 cells was determined (Fig. 5) [22], [23]. As seen in infection with HCVcc (JFH1), expression of ApoA1, ApoA2, ApoC1, ApoC2, ApoC3 and ApoE enhanced the formation of infectious particles of TH/JFH1 and S310/JFH1 chimeric viruses. These results suggest that ApoA1, ApoA2, ApoC1, ApoC2, ApoC3 and ApoE redundantly participate in the efficient formation of infectious HCV particles of genotypes 1b, 2a and 3a.

### Apolipoproteins participate in the post-envelopment step of particle formation

**Figure 6:**
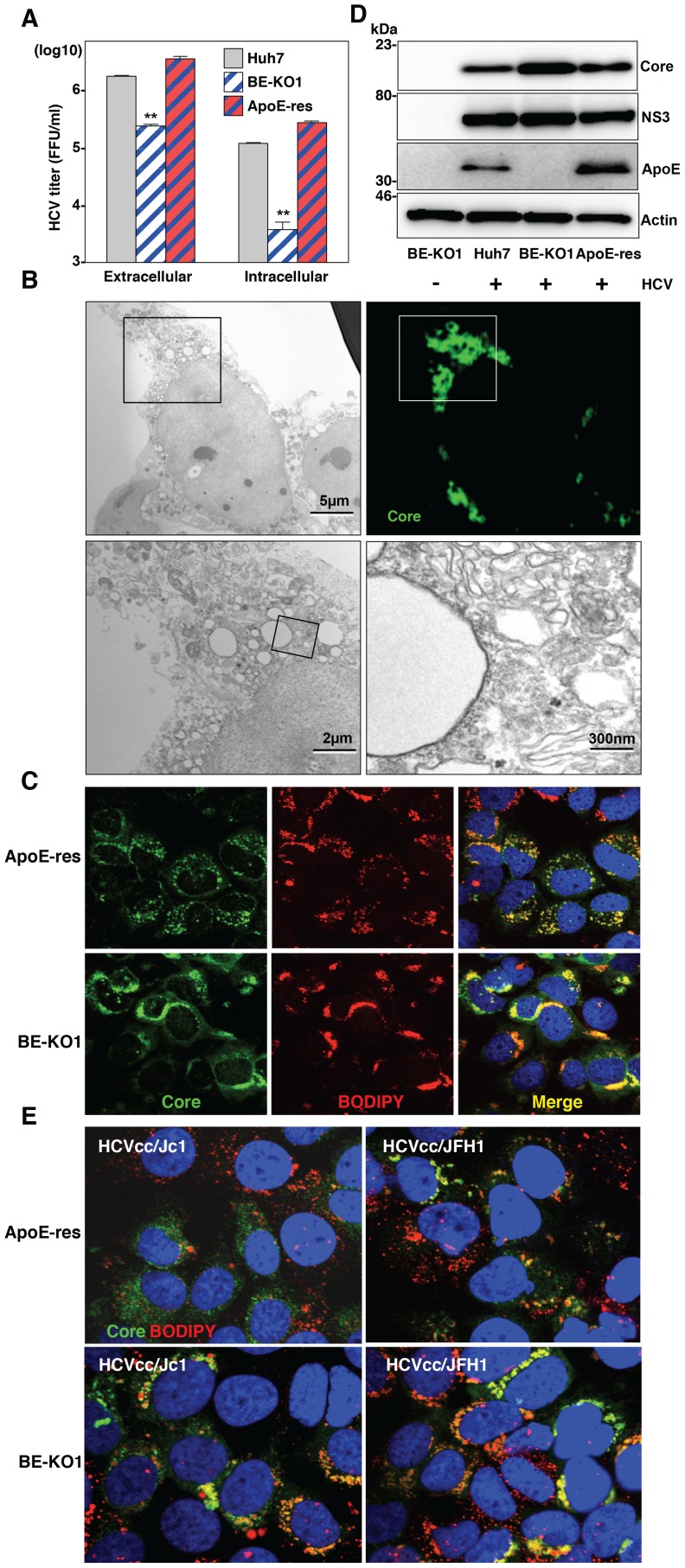
Accumulation of core proteins around lipid droplets in BE-KO1 cells. (A) Extracellular and intracellular infectious titers in Huh7, BE-KO1 and ApoE-restored cells infected by lentiviral vector (ApoE-res) were determined at 72 h post-infection with HCVcc at an MOI of 1 by focus-forming assay. Asterisks indicate significant differences (**, P<0.01) versus the results for parental cells. (B) BE-KO1 cells infected with HCVcc at an MOI of 1 were stained with anti-Core antibody at 72 h post-infection and examined by fluorescence microscopy. Identical fields were observed under electron microscopy by using the correlative FM-EM technique. The boxed areas are magnified and displayed. Huh7, BE-KO1 and ApoE-res cells infected with HCVcc at an MOI of 1 were subjected to immunofluorescence analyses by using anti-Core antibody (C), and immunoblotting by using antibodies against Core, NS3, ApoE, and actin at 72 h post-infection (D). Lipid droplets and cell nuclei were stained by BODIPY and DAPI, respectively. (E) BE-KO1 and ApoE-res cells infected with Jc1 strain-based HCVcc (HCVcc/Jc1; left panel) or JFH1 strain-based HCVcc (HCVcc/JFH1; right panel) at an MOI of 1 were subjected to immunofluorescence analysis by using anti-Core antibody at 72 h post-infection. Lipid droplets and cell nuclei were stained by BODIPY and DAPI, respectively.

To determine the details of the assembly of infectious HCV particles in the BE-KO1 cells, intracellular infectious titers were determined in Huh7, BE-KO1 and ApoE-res cells by using the freeze and thaw method. Not only intracellular but also extracellular infection titers were impaired in BE-KO1 cells compared with those in parental and ApoE-res cells (Fig. 6A), suggesting that intracellular particle formation is impaired by deficiencies in the expression of ApoB and ApoE. Previous reports have shown that the recruitment of viral proteins around LD and redistribution of LD are essential for HCV assembly [24]. To clarify the roles of the exchangeable apolipoproteins on HCV assembly in more detail, we examined the intracellular localization of viral proteins, LD and ER in BE-KO1 and ApoE-res cells. The localization of core proteins around LD and the membranous-web structure forming the replication complex were observed in BE-KO1 cells upon infection with HCVcc, as reported in parental Huh7 cells (Fig. 6B, 6C and Figure S8). However, greater accumulation of core proteins and LD around the perinuclear region was detected in BE-KO1 cells in comparison with ApoE-res cells (Fig. 6C and 6D), supporting the notion that apolipoproteins participate in the infectious particle formation in HCV rather than viral RNA replication. Previous studies revealed that core proteins were mainly localized on the ER membrane upon infection with the genotype 2a Jc1 strain-based HCVcc (HCVcc/Jc1), and inhibition of capsid assembly and envelopment caused accumulation of core proteins on the surface of LD [25]–[27]. In ApoE-res cells, core proteins of HCVcc/Jc1 were mainly localized on the ER membrane, in contrast to the co-localization of core proteins of HCVcc (JFH1) with LD (Fig. 6E upper). However, core proteins were accumulated around LD in BE-KO1 cells infected with HCVcc/Jc1, as seen in those infected with HCVcc (JFH1) (Fig. 6E lower). These results suggest that apolipoproteins participate in the steps of HCV particle formation occurring after HCV protein assembly on the LD.

**Figure 7:**
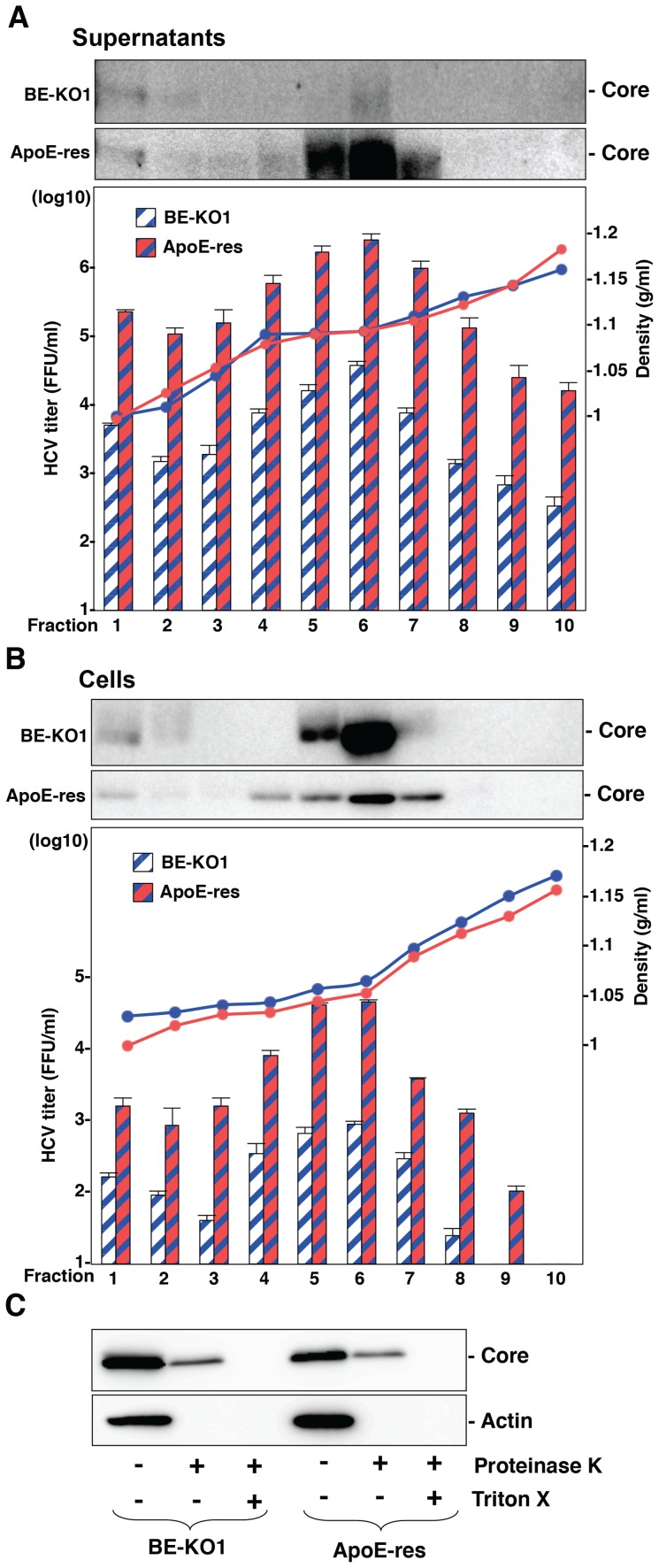
Apolipoproteins participate in the post-envelopment step of the HCV life cycle. The supernatants (A) and lysates (B) of BE-KO1 and ApoE-restored (ApoE-res) cells infected with HCVcc at an MOI of 1 were subjected to density gradient fractionation. Each fraction was subjected to immunoblotting using anti-Core antibody (upper). The infectious titers and densities of each fraction were determined (lower). (C) The lysates of BE-KO1 and ApoE-res cells infected with HCVcc at an MOI of 1 were subjected to proteinase K digestion protection assay. Lysates were separated into 3 parts and incubated for 1 h on ice in the presence or absence of 50 µg/ml proteinase K with/without pretreatment with 5% Triton-X and then subjected to immunoblotting.

To further examine the involvement of apolipoproteins in the infectious particle formation of HCV, culture supernatants and cell lysates of BE-KO1 and ApoE-res cells infected with HCVcc were analyzed by buoyant density ultracentrifugation (Fig. 7A–B) [28]. Secretion of viral capsids in the supernatants was severely impaired in BE-KO1 cells in comparison with that in ApoE-res cells (Fig. 7A upper), in contrast to the detection of abundant intracellular capsids in both cell lines (Fig. 7B upper). Although peak levels of the core proteins and infectious titers were detected around 1.08 g/ml in both cell lines, the infectious titers in all fractions of BE-KO1 cells were significantly lower than those in ApoE-res cells, supporting the notion that apolipoproteins participate in the post-assembly process of HCV capsids which is required to confer infectivity. Next, to examine the involvement of apolipoproteins in the envelopment of HCV particles, lysates of BE-KO1 and ApoE-res cells infected with HCVcc were treated with proteinase K in the presence or absence of Triton X [26]. Protection of HCV core proteins from the protease digestion was observed in both cell lysates (Fig. 7C), suggesting that apolipoproteins are not involved in the envelopment of HCV particles. Collectively, these results suggest that exchangeable apolipoproteins participate in the post-envelopment step of HCV particle formation.

### Amphipathic α-helices in exchangeable apolipoproteins participate in the formation of infectious HCV particles through the interaction with viral particles

**Figure 8:**
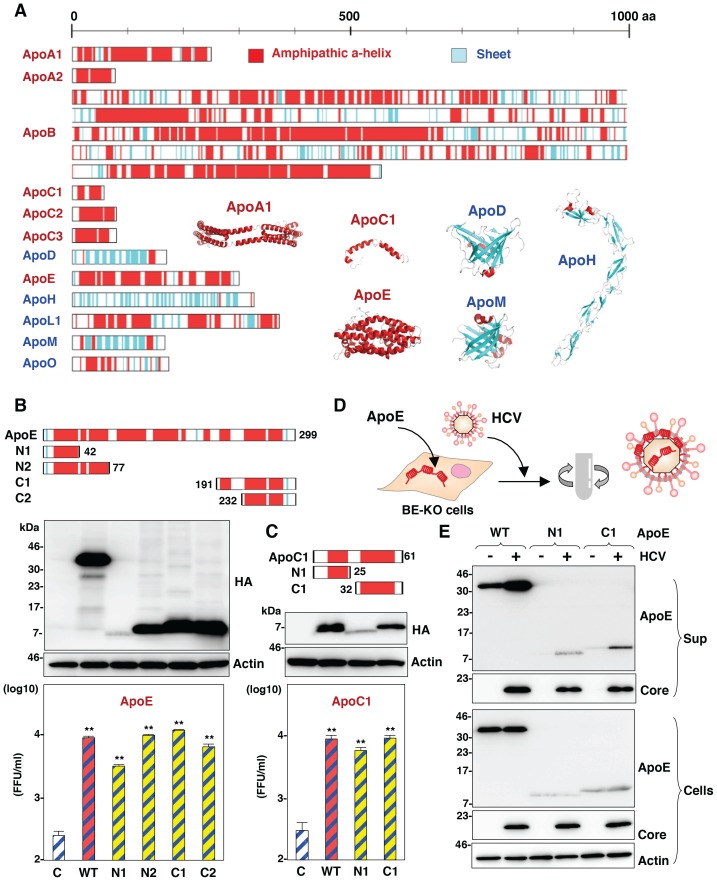
Amphipathic α-helices in apolipoproteins participate in the infectious particle formation of HCV. (A) Predicted or experimentally determined secondary structures of apolipoproteins. Secondary structures of the helices and sheets in the apolipoproteins are colored red and cyan, respectively. The three-dimensional structures of ApoA1 (Protein Data Bank (PDB) ID, 3R2P), ApoC1 (PDB ID, 1IOJ), ApoD (PDB ID, 2HZR), ApoE (PDB ID, 2L7B), ApoH (PDB ID, 1C1Z) and ApoM (PDB ID, 2XKL) are also shown in a ribbon model using the same color code of secondary structures. In cases in which the structure was not available, the secondary structure was predicted by using a CLC Genomics Workbench. (B,C) Schematics of the ApoE- and ApoC1-deletion mutants (upper). Deletion mutants with HA tags expressed in BE-KO1 cells by lentiviral vectors were detected by immunoblotting (middle). BE-KO1 cells expressing the WT or deletion mutants of ApoE or ApoC1 were infected with HCVcc at an MOI of 1, and infectious titers in the culture supernatants were determined by focus-forming assay at 72 h post-infection. Asterisks indicate significant differences (**, P<0.01) versus the results for control cells. (D) Schematic of the concentration of viral particles from HCV-infected cells using ultracentrifugation. (E) BE-KO1 cells expressing the WT or deletion mutants of ApoE were infected with HCVcc at an MOI of 1. Culture supernatants harvested at 72 h post-infection were concentrated by ultracentrifugation at 32,000 rpm for 2 h at 4°C, and subjected to immunoblotting.

To determine the structural relevance of apolipoproteins involved in the HCV assembly, the secondary structures of the apolipoproteins were deduced by using a CLC Genomics Workbench and previous reports (Fig. 8A) [29]–[34]. Tandem repeats of amphipathic α-helices were observed in the apolipoproteins capable of rescuing HCV assembly in BE-KO1 cells, but not in those lacking this activity, suggesting that amphipathic α-helices in the apolipoproteins participate in the assembly of HCV. To examine the involvement of the amphipathic α-helices of the exchangeable apolipoproteins in the particle formation of HCV, we constructed expression plasmids encoding deletion mutants of ApoE and ApoC1, and then these deletion mutants were exogenously expressed in BE-KO1 cells by lentiviral vectors (Fig. 8B and C upper panels). The expression of all of the deletion mutants of ApoE and ApoC1 containing either N-terminal or C-terminal amphipathic α-helices rescued the particle formation of HCV in BE-KO1 cells (Fig. 8B and C lower panels), suggesting that amphipathic α-helices in the apolipoproteins play crucial roles in the production of infectious HCV particles. In addition, more abundant full-length and truncated ApoE were detected in the precipitates of the culture supernatants of cells infected with HCVcc than those of mock-infected cells concentrated by ultracentrifugation, suggesting that the amphipathic α-helices of apolipoproteins are directly associated with HCV particles (Fig. 8D and E). Taken together, the data in this study strongly suggest that exchangeable apolipoproteins redundantly participate in the infectious particle formation of HCV through the interaction between amphipathic α-helices and viral particles.

## Discussion

In this study, we demonstrated the redundant roles of ApoB and the exchangeable apolipoproteins ApoA1, ApoA2, ApoC1, ApoC2, ApoC3 and ApoE in the assembly of infectious HCV particles. The deficiencies of both ApoB and ApoE inhibited the production of infectious HCV particles in Huh7 cells, and exogenous expression of exchangeable apolipoproteins rescued the particle formation. cDNA microarray revealed that the expression patterns of exchangeable apolipoproteins in hepatic cancer cell lines are widely different from those in liver tissue. In previous reports, ApoE and ApoB were identified as important host factors for the assembly of infectious HCV particles [10], [11], and knockdown of ApoE and ApoB expression also inhibited the production of infectious particles in this study. Because ApoB and ApoE are major apolipoproteins in VLDL, several reports have suggested that the VLDL production machinery participates in the production of HCV particles. Furthermore, density gradient analyses revealed co-fractionation of HCV RNA with lipoproteins, with the resulting complexes being termed lipoviroparticles (LVP) [12], [35]. However, it has been reported that there is no correlation between secretion of VLDL and production of LVP [36]. In addition, exogenous expression of ApoE facilitated the infectious particle formation of HCV in 293T cells stably expressing CLDN1 and miR-122 [16], suggesting that ApoE-mediated particle formation is independent from VLDL production. Furthermore, exogenous expression of ApoA1, a major apolipoprotein of HDL, also facilitated the production of HCV particles as shown in Fig. 4E. These data suggest that the roles of the exchangeable apolipoproteins in HCV assembly are independent from the production of VLDL. MTTP plays crucial roles in the lipoprotein formation through the incorporation of triglyceride into growing lipoprotein and secretion of ApoB [21]. Although it has been shown that treatment with an MTTP inhibitor impairs the production of HCV particles [11], in this study, we found that knockout of MTTP abrogated the secretion of ApoB but not the production of infectious HCV particles. Collectively, these data suggest that exchangeable apolipoproteins redundantly participate in the infectious particle formation of HCV independently from lipoprotein secretion machinery.

Production of HCV capsids in the culture supernatants is impaired in 293T cells expressing miR-122 due to lack of ApoE expression, but envelopment of viral capsids is observed [37], suggesting that ApoE is involved in the post-envelopment step. Coller et al. suggested that ApoE is associated with *de novo* formation of HCV particles during secretory pathway based on an experiment using HCV possessing a tetracysteine-tag in the core protein [38]. In this study, ApoA1, ApoA2, ApoC1, ApoC2, ApoC3 and ApoE enhanced the formation of HCV particles in the post-envelopment step. These results suggest that a direct interaction between exchangeable apolipoproteins and enveloped particles in the ER lumen facilitates an efficient secretion of infectious HCV particles. Ultrastructural analysis of HCV particles has shown that large amounts of apolipoproteins, including ApoA1, ApoB and ApoE, bind to the surface of viral particles [39]. Interestingly, ApoE-specific antibodies were more efficient in capturing viral particles than α-E1/E2 antibodies, and significantly large numbers of gold particles reacting with ApoE were observed per virion than those with E2, suggesting that viral envelope proteins are masked by a large amount of apolipoproteins. The unique characteristics of interaction between apolipoproteins and HCV particles might be applied for visualization of entry and purification of HCV particles by using GFP- or affinity-tagged amphipathic α-helices of apolipoproteins. In the previous report, virocidal amphipathic helical peptides impaired the infectivity of viral particles [40]. There is a possibility that such peptide influences on the interaction between apolipoproteins and viral particles, and might be a new therapeutic approach.

In previous reports, the importance of the interaction between lipoprotein receptors and ApoE in the entry of HCV has been well established. Lipoprotein receptors including scavenger receptor class B type 1 (SR-B1) and low-density lipoprotein receptor (LDLR) are involved in HCV entry into the target cells [41], [42]. LDLR is thought to mediate cell attachment of HCV through an interaction with virus associated ApoE [43], [44]. SR-B1 also interacts with ApoE and hypervariable region 1 (HVR1) in the envelope protein of HCV [43]. In this study we have shown that exchangeable apolipoproteins including not only ApoE but also ApoA and ApoC facilitate the production of infectious HCV particles, and that exchangeable apolipoproteins directly associate with viral particles. Meunier et al. reported that ApoC1 associates intracellularly with viral particles during particle morphogenesis and enhances the entry of HCV through an interaction of the C-terminal region of ApoC1 with heparan sulfate [45]. Another group also showed that ApoC1 enhances HCV infection through the triple interplay among HVR1, ApoC1, and SR-B1 [46]. These results suggest that the interaction of HCV particles with apolipoproteins also participates in the entry through the binding of lipoprotein receptors including SR-B1 and LDLR.

Although the gene-knockout technique is essential to obtain reproducible and reliable data, and many knockout mice have been produced in various research areas, the development of experimental tools for HCV study has also been hampered by the narrow cell tropism [47], [48]. A humanized mouse model in which human liver cells were xenotransplanted into immunodeficient mouse was developed and provided an important platform for the analysis of pathogenesis and the development of antivirals for HCV [49]. However, the exogenous expression of human receptor molecules required for HCV entry and impairment of innate immunity are required for the complete propagation of HCV in mice [50]. Gene-knockout techniques using a CRISPR/Cas9 system composed of guide RNA and Cas9 nuclease that form RNA-protein complexes to cleave the target sequences [19] have allowed quick and easy establishment of gene-knockout mice and cancer cell lines [51], [52], and indeed, such MTTP-knockout cell lines were established also in this study. Recently, the high-throughput screening of host factors involved in several conditions was reported by using a CRISPR/Cas9 system [53]. Together, these novel genome-editing techniques are expected to reveal the precise roles of host factors involved in the HCV life cycle.

In summary, we have shown that apolipoproteins, including ApoA1, ApoA2, ApoC1, ApoC2, ApoC3, ApoE and ApoB, possess redundant roles in the assembly of HCV through the interaction of the amphipathic α-helices in the apolipoproteins with viral particles in the post-envelopment step. It is hoped that these findings will provide clues to the life cycle of HCV and assist in the development of novel antivirals targeting the assembly process of HCV.

## Materials and Methods

### NextBio Body Atlas

The NextBio Body Atlas application presents an aggregated analysis of gene expression across various normal tissues, normal cell types, and cancer cell lines [20]. It enables us to investigate the expression of individual genes as well as gene sets. Samples for Body Atlas data are obtained from publicly available studies that are internally curated, annotated, and processed. Body Atlas measurements are generated from all available RNA expression studies that used Affymetrix U133 Plus or U133A Genechip Arrays for human studies. The results from 128 human tissue samples were incorporated from 1,067 arrays; 157 human cell types from 1,474 arrays; and 359 human cancer cell lines from 376 arrays. Gene queries return a list of relevant tissues or cell types rank-ordered by absolute gene expression and grouped by body systems or across all body systems. In the current analysis, we determined the expression levels of the apolipoproteins ApoA1, ApoA2, ApoB, ApoC1, ApoC2, ApoC3, ApoD, ApoE, ApoH, ApoL1, ApoL2 and ApoO in liver tissue. We used an analysis protocol developed by NextBio, the details of which have been described previously [20].

### cDNA microarray

Expression profiling was generated using the 4 x 44 K whole human genome oligo-microarray ver.2.0 G4845A (Agilent Technologies) as previously described [54]. Raw data were imported into Subio platform ver.1.12 (Subio) for database management and quality control. Raw intensity data were normalized against GAP-DH expression levels for further analysis. These raw data have been accepted by GEO (a public repository for microarray data, aimed at storing MIAME). Access to data concerning this study may be found under GEO experiment accession number GSE32886.

### Cell lines

All cell lines were cultured at 37°C under the conditions of a humidified atmosphere and 5% CO_2_. The human hepatocellular carcinoma-derived Huh7 and human embryonic kidney-derived 293T cells were obtained from Japanese Collection of Research Bioresources (JCRB) Cell Bank (JCRB0403 and JCRB9068), and maintained in DMEM (Sigma) supplemented with 100 U/ml penicillin, 100 µg/ml streptomycin, and 10% fetal calf serum (FCS). The Huh7-derived cell line Huh7.5.1 was kindly provided by F. Chisari. Huh7 cells harboring JFH1-based HCV-SGR were prepared according to the method of a previous report [54] and maintained in DMEM containing 10% FCS and 1 mg/ml G418 (Nakalai Tesque).

### Plasmids

The cDNA clones of pri-miR-122, ApoA1, ApoA2, ApoC1, ApoC2, ApoC3, ApoE, ApoH, and AcGFP were inserted between the XhoI and XbaI sites of lentiviral vector pCSII-EF-RfA, which was kindly provided by M. Hijikata, and the resulting plasmids were designated pCSII-EF-miR-122, pCSII-EF-MT-apolipoproteins, and pCSII-EF-AcGFP, respectively. The deletion mutants of ApoC1 and ApoE were amplified by PCR and introduced into pCSII-EF. pHH-JFH1-E2p7NS2mt contains three adaptive mutations in pHH-JFH1 [55]. The pFL-J6/JFH1 plasmid that encodes the entire viral genome of the chimeric strain of HCV-2a, J6/JFH1, was kindly provided by Charles M. Rice [8]. pTH/JFH1 (genotype 1b) and pS310/JFH1 (genotype 3a) were used for the production of chimeric viruses [22], [23]. The plasmid pX330, which encodes hCas9 and sgRNA, was obtained from Addgene (Addgene plasmid 42230). The fragments of guided RNA targeting the MTTP gene were inserted into the Bbs1 site of pX330 and designated pX330-MTTP. The plasmids used in this study were confirmed by sequencing with an ABI 3130 genetic analyzer (Life Technologies).

### Antibodies

Mouse monoclonal antibodies to HCV core, β-actin and Calnexin were purchased from Thermo Scientific and Sigma Aldrich, respectively. Mouse anti-ApoA1, ApoB, ApoC1, ApoE and ApoH antibodies were purchased from Cell Signaling, ALerCHEK Inc., Abnova, NOVUS Biologicals, and Santa Cruz Biotechnology, respectively. Rat anti-ApoA2 and Sheep anti-ApoC2 antibodies were purchased from R&D systems. Rabbit anti-NS5A antibody was prepared as described previously [54]. Alexa Fluor (AF) 488-conjugated anti-rabbit or mouse IgG antibodies, and AF594-conjugated anti-mouse IgG2a antibodies were purchased from Life Technologies.

### Gene silencing

A small interfering RNA (siRNA) pool targeting various apolipoproteins (siGENOME SMARTpool) and control nontargeting siRNA were purchased from Dharmacon, and transfected into cells using Lipofectamine RNAi MAX (Life Technologies) according to the manufacturer's protocol. A human shRNA library was purchased from Takara Bio Inc.

### Preparation of viruses

Upon transfection of pHH-JFH1-E2p7NS2mt or *in vitro* transcribed TH/JFH1, J6/JFH1 and S310/JFH1 RNA into Huh7.5.1 cells, HCV in the supernatant was collected after serial passages, and infectious titers were determined by a focus-forming assay and expressed in focus-forming units (FFU) [22], [23], [54]. To compare the localization of core protein, J6/JFH1 was used in Fig. 6E. Pseudoparticles expressing HCV envelope glycoprotein were generated in 293T cells as previously reported [5], and infectivity was assessed by luciferase expression using the Bright-Glo Luciferase assay system (Promega) and expressed in relative light units (RLU).

### Lipofection and lentiviral gene transduction

The lentiviral vectors and ViraPower Lentiviral Packaging Mix (Life Technologies) were co-transfected into 293T cells by Trans IT LT-1 (Mirus), and the supernatants were recovered at 48 h post-transfection. The lentivirus titer was determined by the Lenti-XTM qRT-PCR Titration Kit (Clontech), and the expression levels and AcGFP were determined at 48 h post-inoculation.

### Immunoblotting

Cells lysed on ice in lysis buffer (20 mM Tris-HCl [pH 7.4], 135 mM NaCl, 1% Triton-X 100, 10% glycerol) supplemented with a protease inhibitor mix (Nacalai Tesque) were boiled in loading buffer and subjected to 5–20% gradient SDS-PAGE. The proteins were transferred to polyvinylidene difluoride membranes (Millipore) and reacted with the appropriate antibodies. The immune complexes were visualized with SuperSignal West Femto Substrate (Pierce) and detected by the LAS-3000 image analyzer system (Fujifilm).

### Generation of gene-knockout Huh7 cell lines

Custom ZFN plasmids were designed to bind and cleave the ApoB, ApoE and MTTP genes and were obtained from Sigma Aldrich. Huh7 cells were transfected with *in vitro* transcribed ZFNs mRNA or pX330-MTTP by Lipofectamine 2000 (Life Technologies), and single cell clones were established by the single cell isolation technique. To screen for gene-knockout Huh7 cell clones, mutations in target loci were determined by using a Surveyor assay as previously described [56]. Frameshift of the genes and deficiencies of protein expression were confirmed by direct sequencing and immunoblotting analysis, respectively.

### Enzyme-linked immunosorbent assay (ELISA)

Protein concentrations of ApoB or ApoE in the culture supernatants were determined by using ELISA immunoassay kits (Alercheck Inc.) according to the manufacturer's protocol.

### Quantitative RT-PCR

Total RNA was extracted from cells by using an RNeasy minikit (Qiagen) and the first-strand cDNA synthesis and qRT-PCR were performed with TaqMan EZ RT-PCR core reagents and a ViiA7 system (Life Technologies), respectively, according to the manufacturer's protocol. The primers for TaqMan PCR targeted to the noncoding region of HCV RNA were synthesized as previously reported [54]. Taqman Gene expression assays were used as the primers and probes targeting to apolipoproteins (Life Technologies). Fluorescent signals were analyzed with the ViiA7 system.

### Immunofluorescence assay

Cells cultured on glass slides were fixed with 4% paraformaldehyde (PFA) in phosphate buffered saline (PBS) at room temperature for 30 min, permeabilized for 20 min at room temperature with PBS containing 0.2% Triton after being washed three times with PBS, and blocked with PBS containing 2% FCS for 1 h at room temperature. The cells were incubated with PBS containing the appropriate primary antibodies at room temperature for 1 h, washed three times with PBS, and incubated with PBS containing AF488- or AF594-conjugated secondary antibodies at room temperature for 1 h. For lipid-droplet staining, cells incubated in medium containing 20 µg/ml BODIPY for 20 min at 37°C were washed with pre-warmed fresh medium and incubated for 20 min at 37°C. Cell nuclei were stained with DAPI. Cells were observed with a FluoView FV1000 laser scanning confocal microscope (Olympus).

### *In vitro* transcription, RNA transfection, and colony formation

The plasmid pSGR-JFH1 was linearized with XbaI, and treated with mung bean exonuclease. The linearized DNA was transcribed *in vitro* by using the MEGAscript T7 kit (Life Technologies) according to the manufacturer's protocol. The *in vitro* transcribed RNA (10 µg) was electroporated into Huh7 cells at 10^7^ cells/0.4 ml under conditions of 190 V and 975 µF using a Gene Pulser (Bio-Rad) and plated on DMEM containing 10% FCS. The medium was replaced with fresh DMEM containing 10% FCS and 1 mg/ml G418 at 24 h post-transfection. The remaining colonies were cloned by using a cloning ring (Asahi Glass) or fixed with 4% PFA and stained with crystal violet at 4 weeks post-electroporation.

### Intracellular infectivity

Intracellular viral titers were determined according to a method previously reported [10]. Briefly, cells were extensively washed with PBS, scraped, and centrifuged for 5 min at 1000× *g*. Cell pellets were resuspended in 500 µl of DMEM containing 10% FCS and subjected to three cycles of freezing and thawing using liquid nitrogen and a thermo block set to 37°C. Cell lysates were centrifuged at 10,000× *g* for 10 min at 4°C to remove cell debris. Cell-associated infectivity was determined by a focus-forming assay.

### Electron microscopy and correlative FM-EM analysis

Correlative fluorescence microscopy-electron microscopy (FM-EM) allows individual cells to be examined both in an overview with fluorescence microscopy and in a detailed subcellular-structure view with electron microscopy. Cells infected with HCVcc were examined by the correlative FM-EM method as described previously [57].

### Buoyant density fractionation

Culture supernatants of cells infected with HCVcc were concentrated 50 times by using Spin-X UF concentrators (Corning), and the intracellular proteins collected after freeze-and-thaw were applied to the top of a linear gradient formed from 10–40% OptiPrep (Axis-Shield) in PBS and spun at 32,000 rpm for 16 h at 4°C by using an SW41 Ti rotor (Beckman Coulter). Aliquots of 10 consecutive fractions were collected, and the infectious titer and density were determined.

### Proteinase K digestion protection assay

The proteinase K digestion protection assay was performed as described previously [37]. Briefly, cells were extensively washed with PBS, scraped, and centrifuged for 5 min at 1000× *g*. The cell pellets were resuspended in 500 µl of PBS and subjected to three cycles of freezing and thawing using liquid nitrogen and a thermo block set to 37°C. The cell lysates were centrifuged at 10,000× *g* for 10 min at 4°C to remove cell debris. The cell lysates were then incubated with 50 µg/ml proteinase K (Life Technologies) in the presence or absence of 5% Triton-X for 1 h on ice, and the digestion was terminated by addition of PMSF (Wako Chemical Industries).

### Statistics

The data for statistical analyses are the average of three independent experiments. Results were expressed as the means ± standard deviation. The significance of differences in the means was determined by Student's *t*-test.

## Supporting Information

Figure S1 **Establishment of ApoB- or ApoE-knockout Huh7 cell lines.** Target sequences of ZFNs to ApoB (A) and ApoE (B) are indicated by red characters inside a red box at the top of the panel. Gene knockout by the sequence modification in the 2 alleles of the ApoB (A) or ApoE (B) gene in knockout cell lines (B-KO1 and B-KO2, or E-KO1 and E-KO2) is shown. Deletion and insertion of the sequences are indicated by dotted lines and blue characters in brackets, respectively. Absence of the expressions of ApoB (C) and ApoE (D) in the knockout cell lines was confirmed by immunoblotting using anti-ApoB and -ApoE antibodies. Expression of ApoB (E) and ApoE (F) in the culture supernatants of 293T, Huh7 and the knockout cell lines was determined by ELISA.
(TIF)

Figure S2 **Both ApoB and ApoE are involved in the formation of infectious HCV particles.** (A) HCVpp were inoculated into Huh7, B-KO1, B-KO2, E-KO1 and E-KO2 cells, and luciferase activities were determined at 48 h post-infection. (B) A subgenomic HCV RNA replicon of the JFH1 strain was electroporated into Huh7, B-KO1 and E-KO1 cells, and colonies were stained with crystal violet at 31 days post-electroporation after selection with 400 µg/ml of G418. HCVcc were inoculated into Huh7, B-KO1, B-KO2, E-KO1 and E-KO2 cells at an MOI of 1 and intracellular HCV RNA at 12, 24, 36 and 60 h post-infection (C), and infectious titers in the culture supernatants at 72 h post-infection (D) were determined by qRT-PCR and focus-forming assay, respectively. (E) Exogenous expression of ApoE in E-KO1 and E-KO2 cells by lentiviral vector was determined by immunoblotting analysis (upper), and infectious titers in the culture supernatants of cells infected with HCVcc at an MOI of 1 were determined at 72 h post-infection by focus-forming assay (lower).
(TIF)

Figure S3 **Establishment of ApoB and ApoE double-knockout (BE-KO) Huh7 cell lines.** Gene knockout by the ZFN in the 2 alleles of the ApoB and ApoE genes in the double-knockout cell lines, BE-KO1 (A) and BE-KO2 (B), is shown. Deletion and insertion of the sequences are indicated by dotted lines and blue characters in brackets, respectively. (C) The absence of the expressions of ApoB and ApoE in BE-KO1 and BE-KO2 was confirmed by immunoblotting using anti-ApoB and -ApoE antibodies. Expression of ApoB (D) and ApoE (E) in the culture supernatants of 293T, Huh7, BE-KO1 and BE-KO2 cells was determined by ELISA.
(TIF)

Figure S4 **Establishment of MTTP-knockout (M-KO) and ApoE and MTTP double-knockout (EM-KO) Huh7 cell lines.** (A) Gene knockout by the ZFN in the 2 alleles of the MTTP gene in the knockout cell lines, M-KO1 and M-KO2, is shown. (B) Expression of MTTP in Huh7, M-KO1 and M-KO2 cells was determined by immunoblotting. Expression of ApoB (C) and ApoE (D) in the culture supernatants of Huh7, M-KO1, M-KO2 and 293T cells was determined by ELISA. (E) Gene knockout in the 2 alleles of the MTTP genes by the CRISPR/Cas9 system and in one allele of the ApoE gene by the ZFN in the double-knockout cell lines, EM-KO1 and EM-KO2, is shown. (F) Expression of MTTP in Huh7, EM-KO1 and EM-KO2 cells was determined by immunoblotting. Expression of ApoB (G) and ApoE (H) in the culture supernatants of Huh7, EM-KO1, EM-KO2 and 293T cells was determined by ELISA. (I) Expression of ApoB mRNA in Huh7, M-KO1, M-KO2, EM-KO1, EM-KO2 and 293T cells was determined by qRT-PCR.
(TIF)

Figure S5 **Gene silencing of apolipoproteins.** BE-KO1 cells infected with HCVcc at an MOI of 1 at 6 h post-transfection with siRNAs targeting ApoA1, ApoA2, ApoC1, ApoC2, ApoC3 and ApoH, and the expression levels of these apolipoproteins were determined by q-RT PCR using specific primers and probes.
(TIF)

Figure S6 **ApoD, ApoL1, and ApoO do not participate in the formation of infectious HCV particles.** Exogenous expression of ApoD, ApoE, ApoL1 and ApoO in BE-KO1 cells by lentiviral vector was determined by immunoblotting analysis (upper), and infectious titers in the culture supernatants of cells infected with HCVcc at an MOI of 1 were determined at 72 h post-infection by focus-forming assay (lower).
(TIF)

Figure S7 **BE-KO1 cells permit propagation of JEV and DENV.** Huh7, BE-KO1 and ApoE-restored (ApoE-res) cells were infected with JEV and DENV at an MOI of 0.1, and infectious titers in the culture supernatants were determined by focus-forming assay at 48 h post-infection.
(TIF)

Figure S8 **Localization of core, NS5A proteins and ER in BE-KO Huh7 cells.** BE-KO1 cells infected with HCVcc at an MOI of 1 were subjected to immunofluorescence analyses by using antibodies against core, NS5A and Calnexin.
(TIF)
