# Supplementary figures and images for: Amphipathic α-Helices in Apolipoproteins Are Crucial to the Formation of Infectious Hepatitis C Virus Particles
Source: PLoS Pathog. 2014 Dec 11;10(12):e1004534. doi: 10.1371/journal.ppat.1004534 (PMC4263759; doi:10.1371/journal.ppat.1004534)

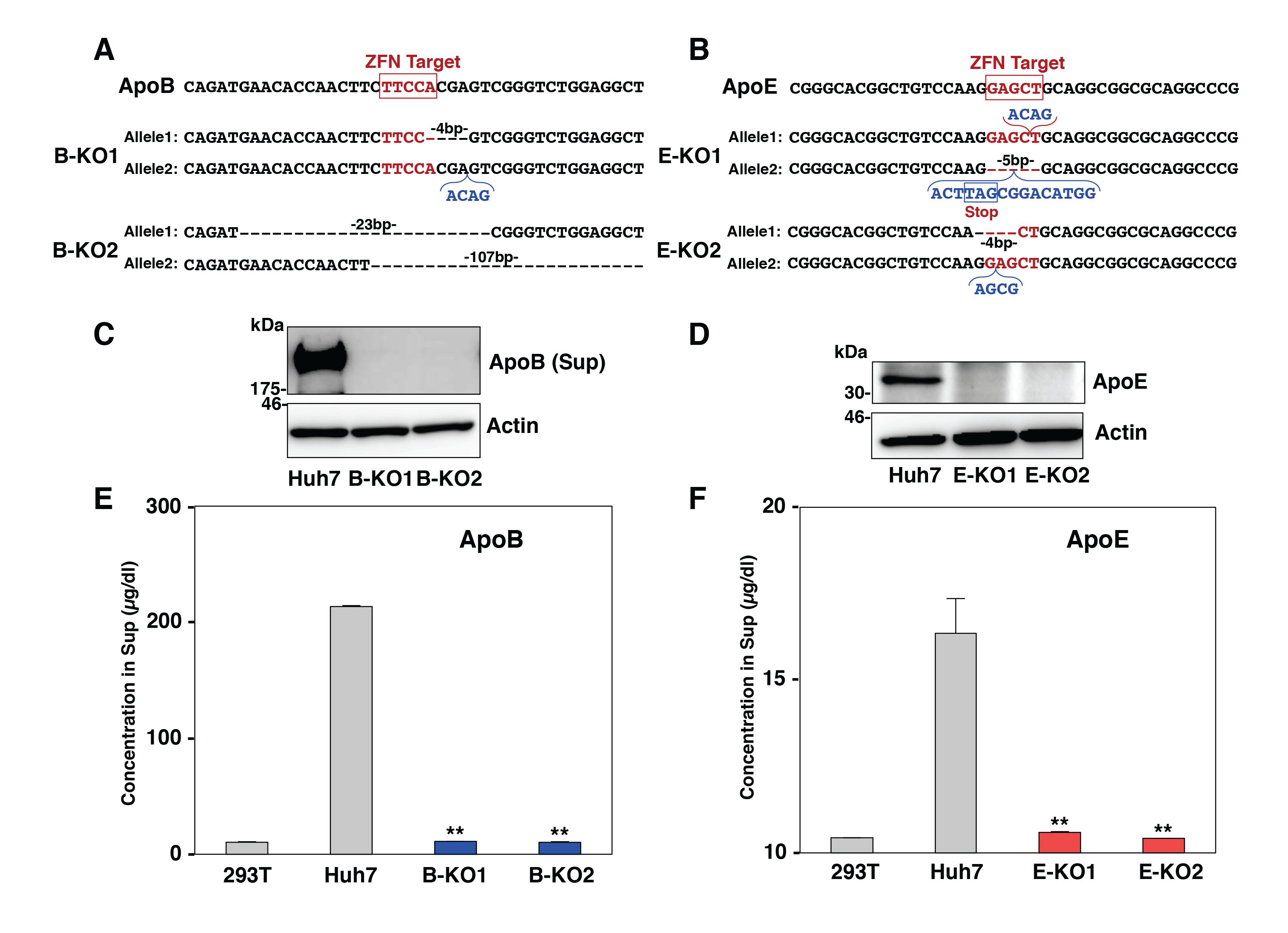

Supplement: Figure S1 — Establishment of ApoB- or ApoE-knockout Huh7 cell lines. Target sequences of ZFNs to ApoB (A) and ApoE (B) are indicated by red characters inside a red box at the top of the panel. Gene knockout by the sequence modification in the 2 alleles of the ApoB (A) or ApoE (B) gene in knockout cell lines (B-KO1 and B-KO2, or E-KO1 and E-KO2) is shown. Deletion and insertion of the sequences are indicated by dotted lines and blue characters in brackets, respectively. Absence of the expressions of ApoB (C) and ApoE (D) in the knockout cell lines was confirmed by immunoblotting using anti-ApoB and -ApoE antibodies. Expression of ApoB (E) and ApoE (F) in the culture supernatants of 293T, Huh7 and the knockout cell lines was determined by ELISA. (TIF) [file ppat.1004534.s001.tif]

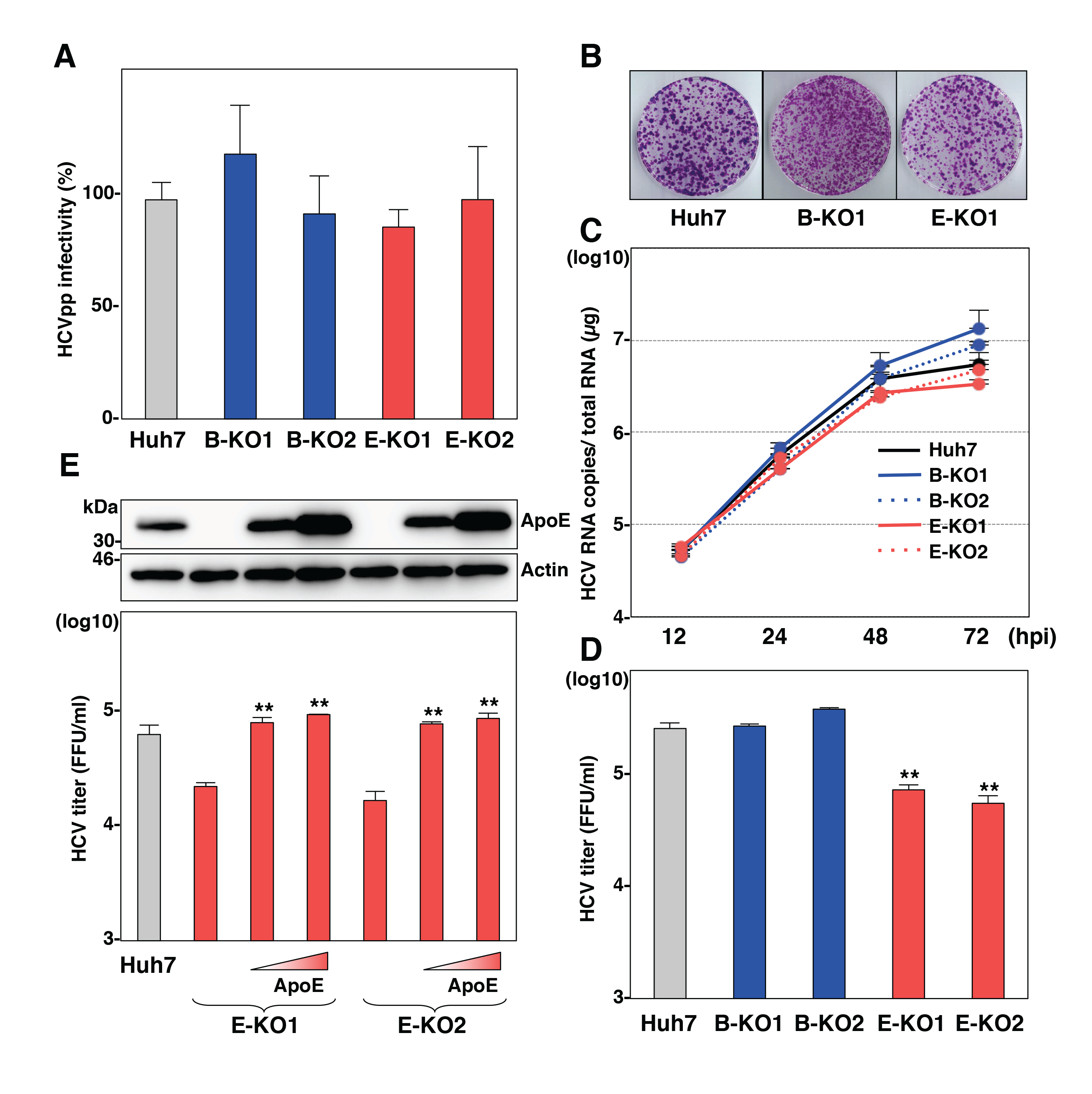

Supplement: Figure S2 — Both ApoB and ApoE are involved in the formation of infectious HCV particles. (A) HCVpp were inoculated into Huh7, B-KO1, B-KO2, E-KO1 and E-KO2 cells, and luciferase activities were determined at 48 h post-infection. (B) A subgenomic HCV RNA replicon of the JFH1 strain was electroporated into Huh7, B-KO1 and E-KO1 cells, and colonies were stained with crystal violet at 31 days post-electroporation after selection with 400 µg/ml of G418. HCVcc were inoculated into Huh7, B-KO1, B-KO2, E-KO1 and E-KO2 cells at an MOI of 1 and intracellular HCV RNA at 12, 24, 36 and 60 h post-infection (C), and infectious titers in the culture supernatants at 72 h post-infection (D) were determined by qRT-PCR and focus-forming assay, respectively. (E) Exogenous expression of ApoE in E-KO1 and E-KO2 cells by lentiviral vector was determined by immunoblotting analysis (upper), and infectious titers in the culture supernatants of cells infected with HCVcc at an MOI of 1 were determined at 72 h post-infection by focus-forming assay (lower). (TIF) [file ppat.1004534.s002.tif]

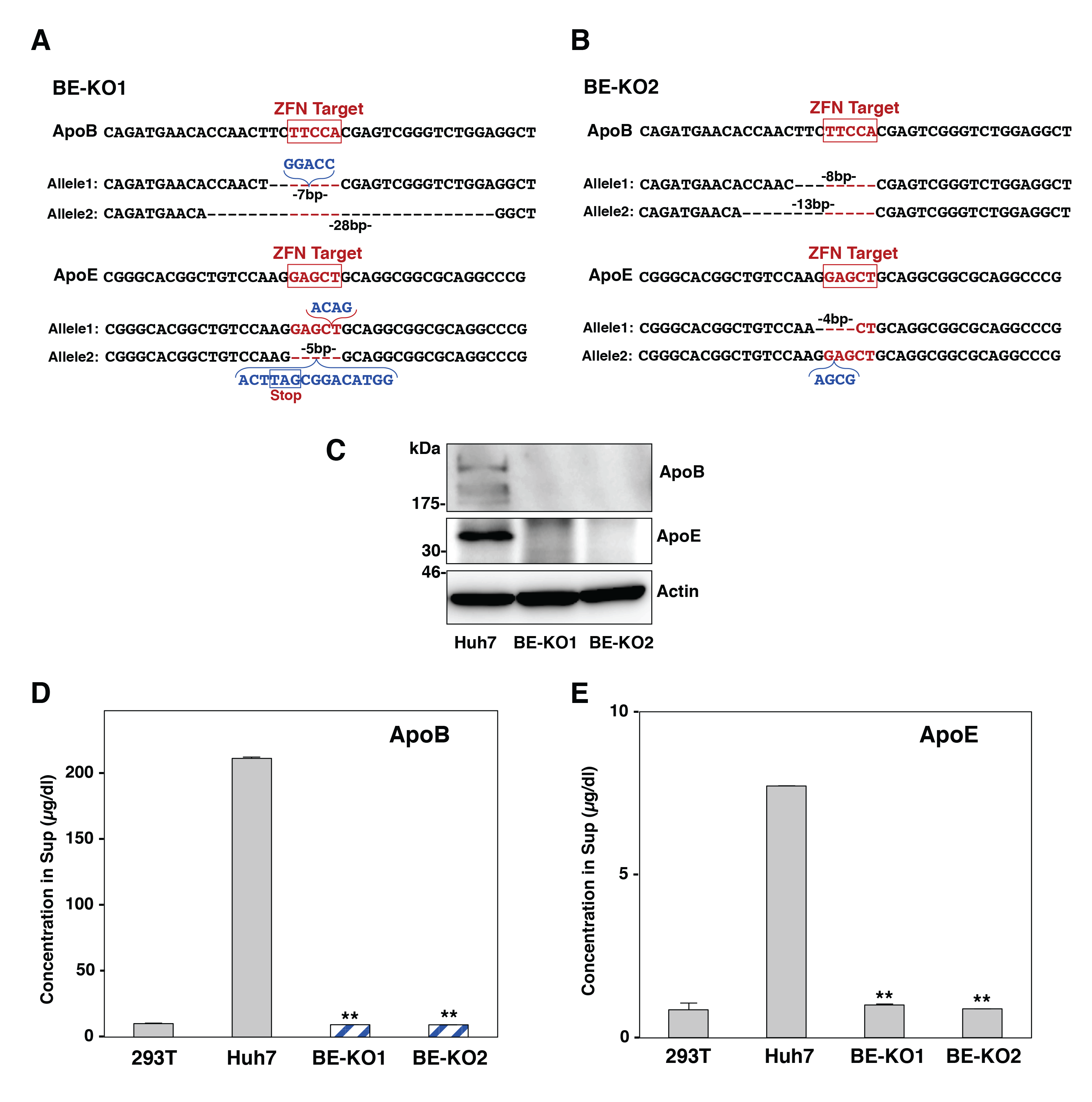

Supplement: Figure S3 — Establishment of ApoB and ApoE double-knockout (BE-KO) Huh7 cell lines. Gene knockout by the ZFN in the 2 alleles of the ApoB and ApoE genes in the double-knockout cell lines, BE-KO1 (A) and BE-KO2 (B), is shown. Deletion and insertion of the sequences are indicated by dotted lines and blue characters in brackets, respectively. (C) The absence of the expressions of ApoB and ApoE in BE-KO1 and BE-KO2 was confirmed by immunoblotting using anti-ApoB and -ApoE antibodies. Expression of ApoB (D) and ApoE (E) in the culture supernatants of 293T, Huh7, BE-KO1 and BE-KO2 cells was determined by ELISA. (TIF) [file ppat.1004534.s003.tif]

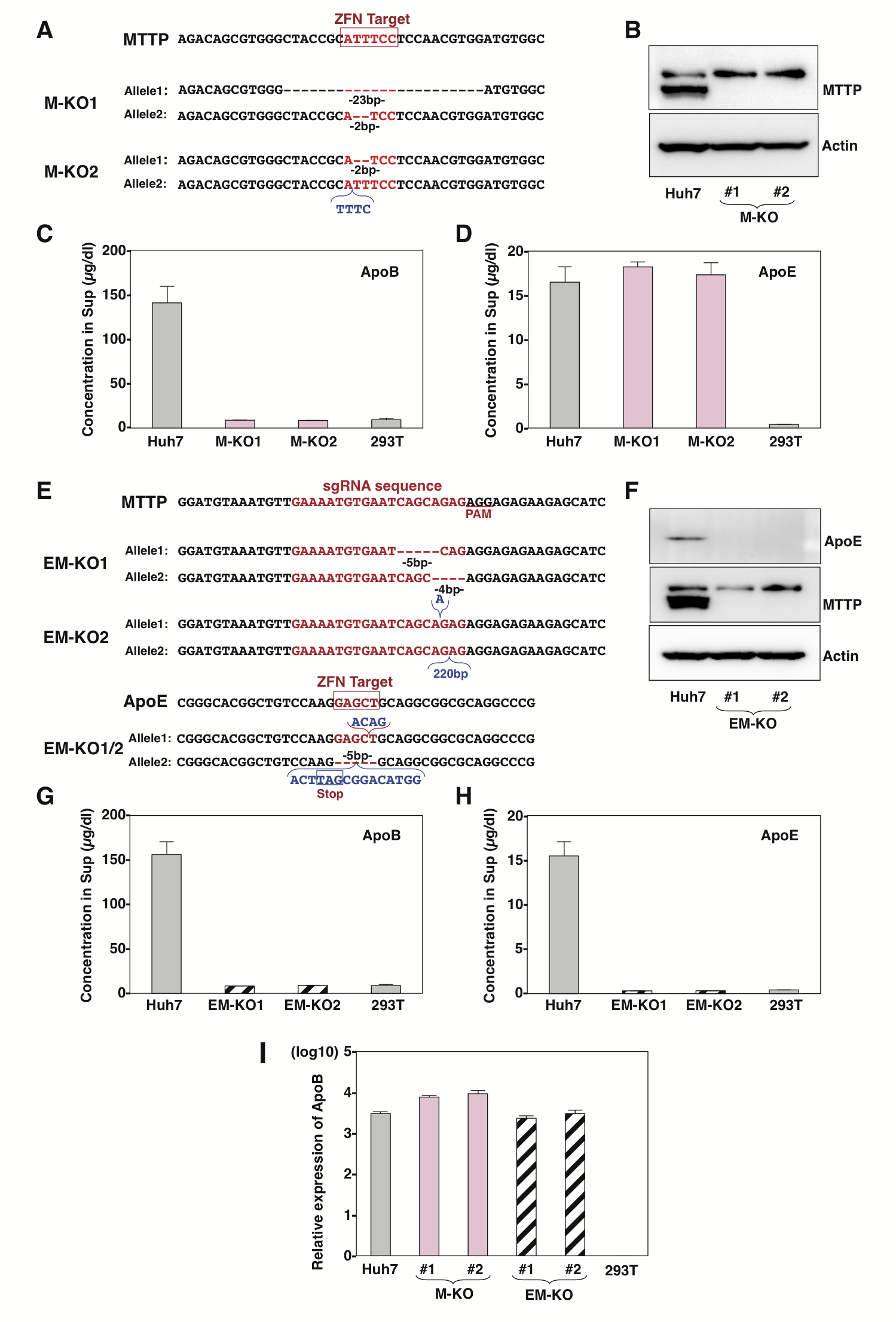

Supplement: Figure S4 — Establishment of MTTP-knockout (M-KO) and ApoE and MTTP double-knockout (EM-KO) Huh7 cell lines. (A) Gene knockout by the ZFN in the 2 alleles of the MTTP gene in the knockout cell lines, M-KO1 and M-KO2, is shown. (B) Expression of MTTP in Huh7, M-KO1 and M-KO2 cells was determined by immunoblotting. Expression of ApoB (C) and ApoE (D) in the culture supernatants of Huh7, M-KO1, M-KO2 and 293T cells was determined by ELISA. (E) Gene knockout in the 2 alleles of the MTTP genes by the CRISPR/Cas9 system and in one allele of the ApoE gene by the ZFN in the double-knockout cell lines, EM-KO1 and EM-KO2, is shown. (F) Expression of MTTP in Huh7, EM-KO1 and EM-KO2 cells was determined by immunoblotting. Expression of ApoB (G) and ApoE (H) in the culture supernatants of Huh7, EM-KO1, EM-KO2 and 293T cells was determined by ELISA. (I) Expression of ApoB mRNA in Huh7, M-KO1, M-KO2, EM-KO1, EM-KO2 and 293T cells was determined by qRT-PCR. (TIF) [file ppat.1004534.s004.tif]

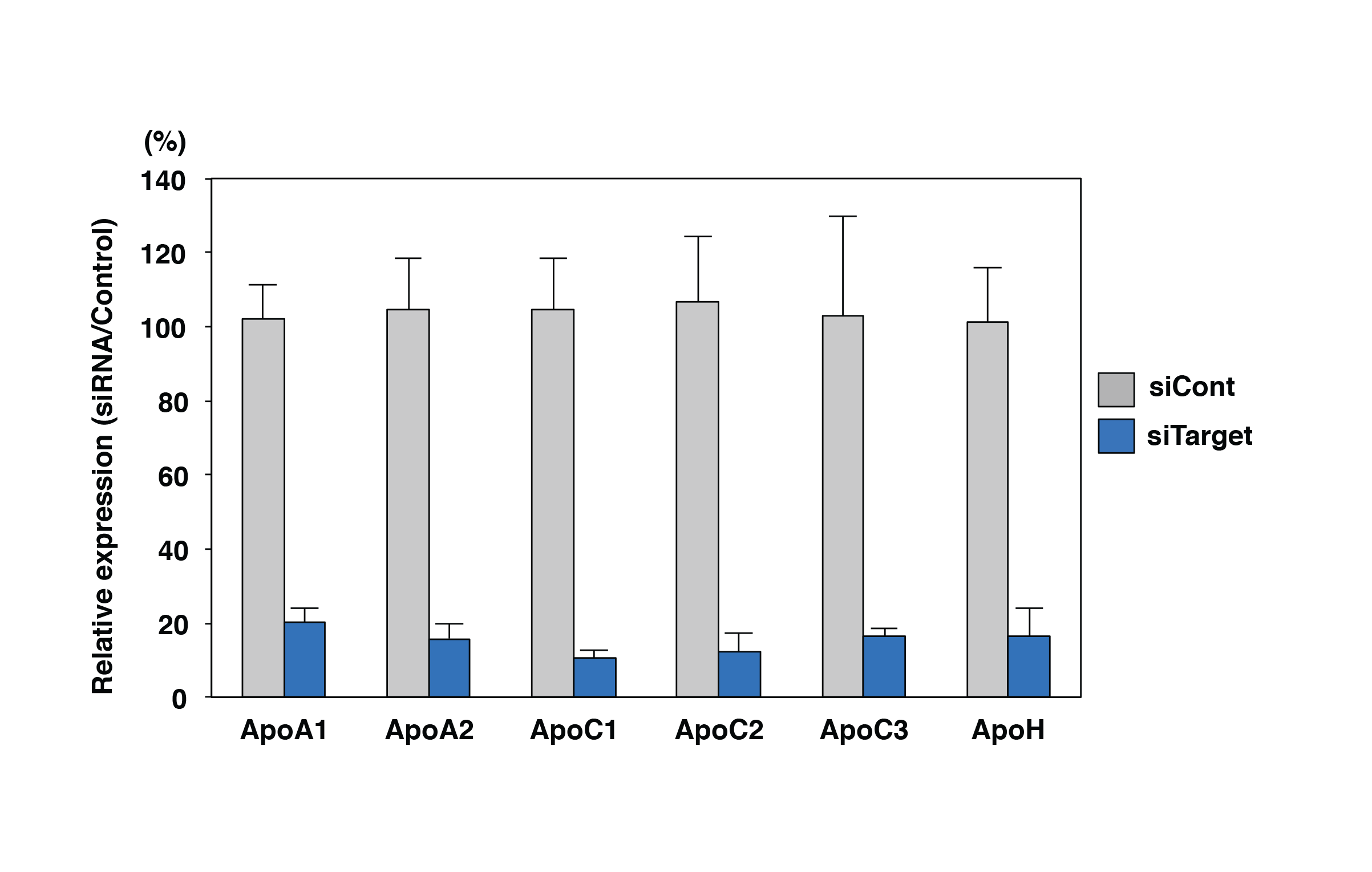

Supplement: Figure S5 — Gene silencing of apolipoproteins. BE-KO1 cells infected with HCVcc at an MOI of 1 at 6 h post-transfection with siRNAs targeting ApoA1, ApoA2, ApoC1, ApoC2, ApoC3 and ApoH, and the expression levels of these apolipoproteins were determined by q-RT PCR using specific primers and probes. (TIF) [file ppat.1004534.s005.tif]

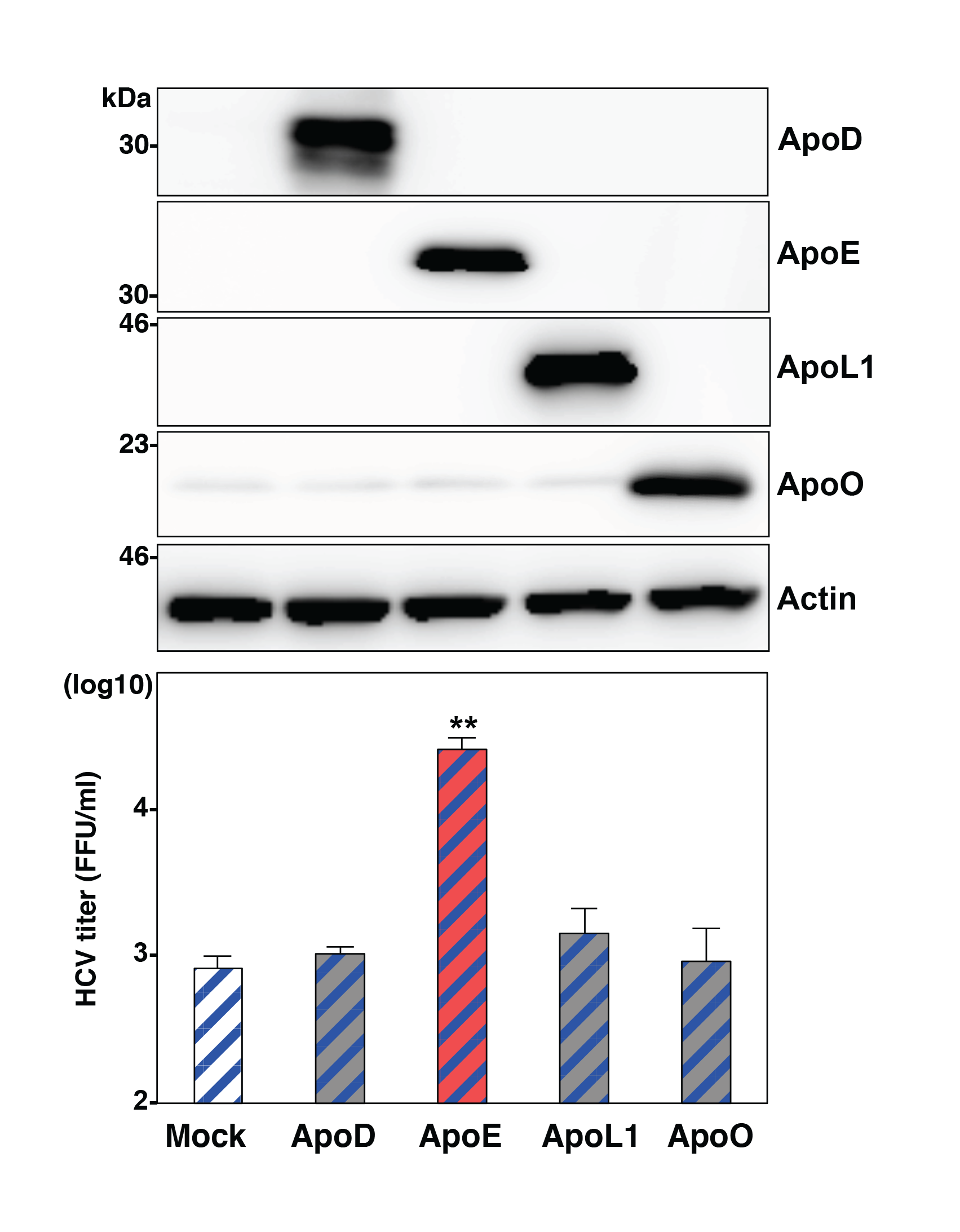

Supplement: Figure S6 — ApoD, ApoL1, and ApoO do not participate in the formation of infectious HCV particles. Exogenous expression of ApoD, ApoE, ApoL1 and ApoO in BE-KO1 cells by lentiviral vector was determined by immunoblotting analysis (upper), and infectious titers in the culture supernatants of cells infected with HCVcc at an MOI of 1 were determined at 72 h post-infection by focus-forming assay (lower). (TIF) [file ppat.1004534.s006.tif]

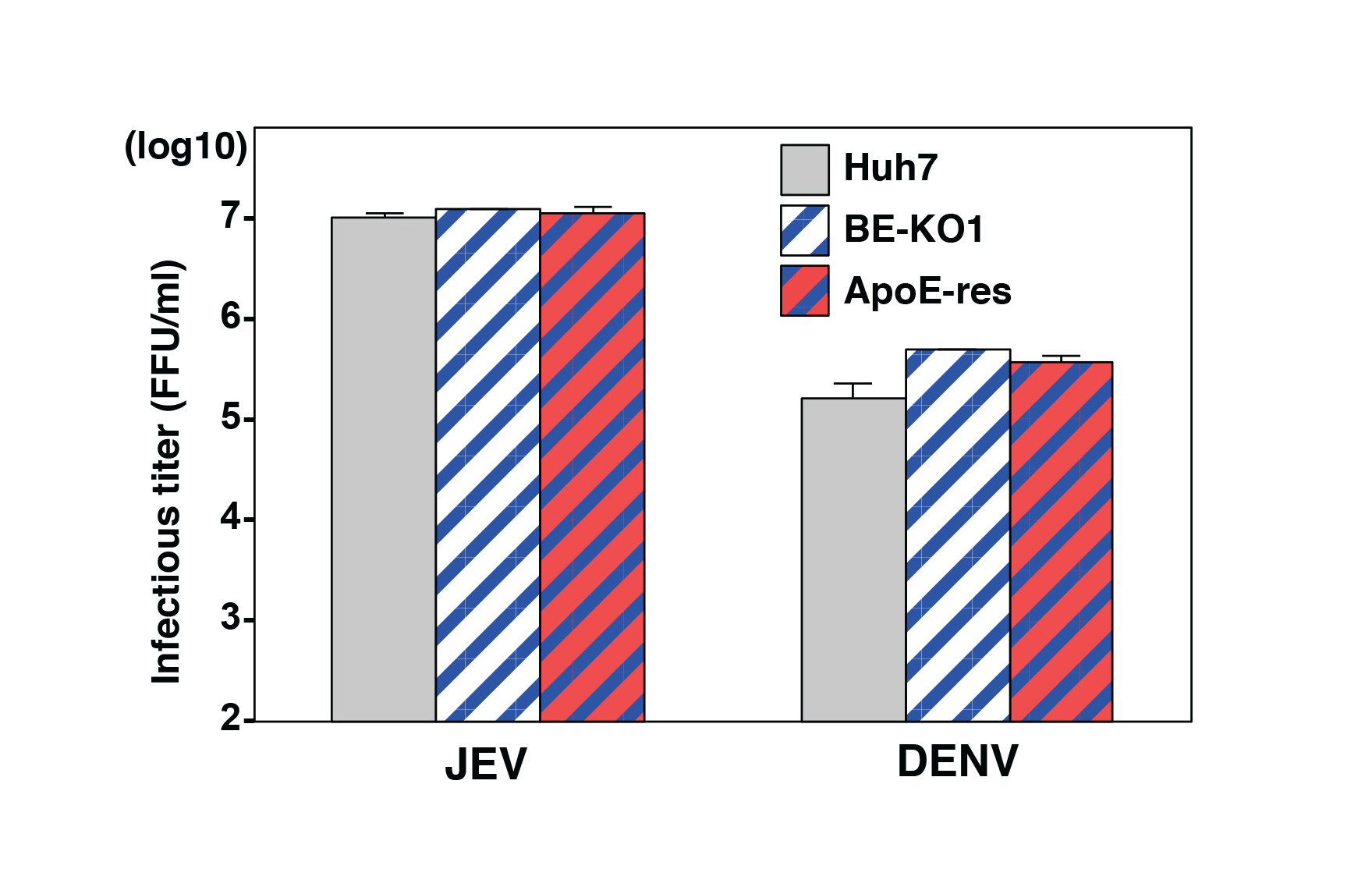

Supplement: Figure S7 — BE-KO1 cells permit propagation of JEV and DENV. Huh7, BE-KO1 and ApoE-restored (ApoE-res) cells were infected with JEV and DENV at an MOI of 0.1, and infectious titers in the culture supernatants were determined by focus-forming assay at 48 h post-infection. (TIF) [file ppat.1004534.s007.tif]

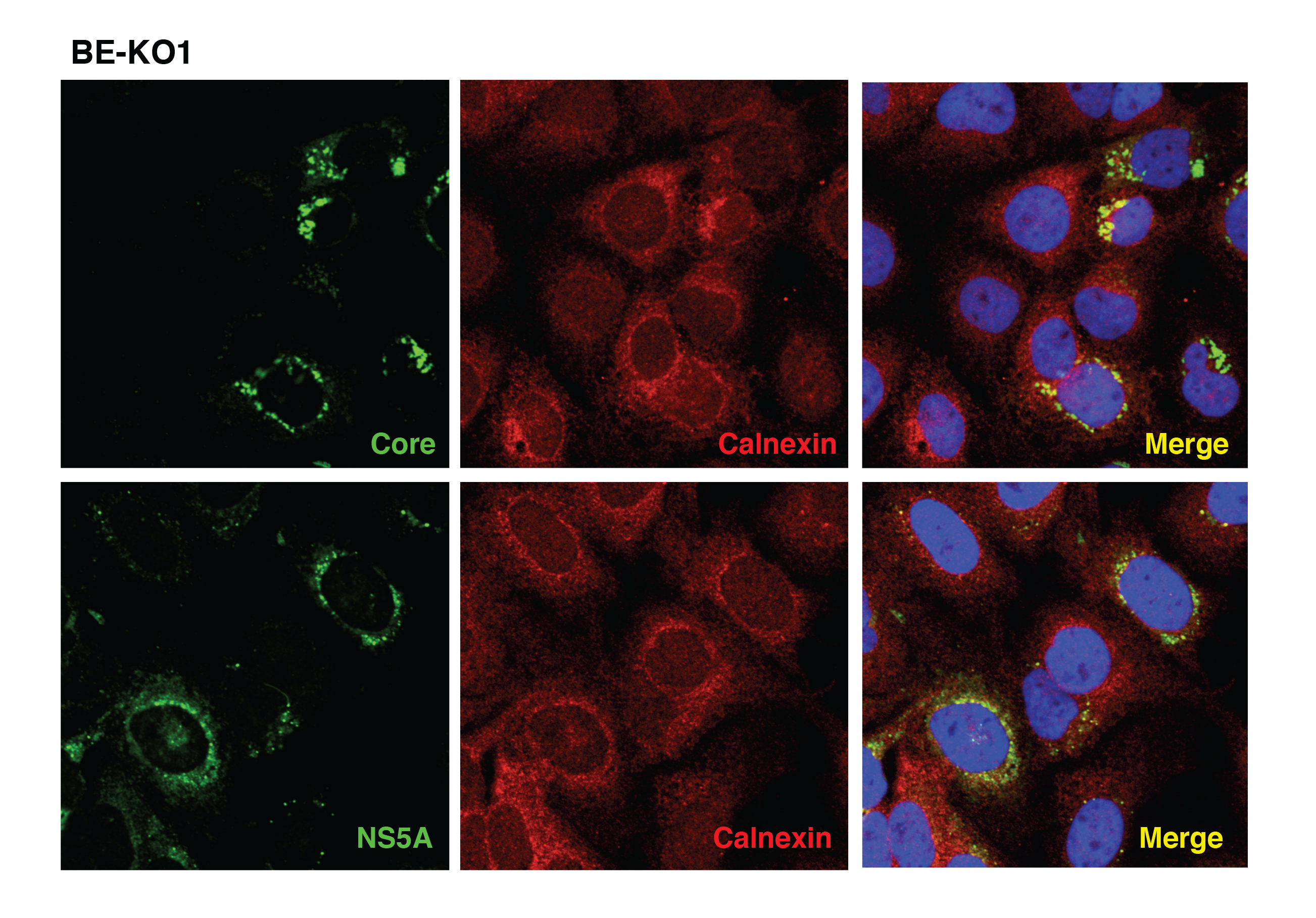

Supplement: Figure S8 — Localization of core, NS5A proteins and ER in BE-KO Huh7 cells. BE-KO1 cells infected with HCVcc at an MOI of 1 were subjected to immunofluorescence analyses by using antibodies against core, NS5A and Calnexin. (TIF) [file ppat.1004534.s008.tif]
